# Genome-wide association study of the candidate genes for grape berry shape-related traits

**DOI:** 10.1186/s12870-022-03434-x

**Published:** 2022-01-20

**Authors:** Chuan Zhang, Liwen Cui, Jinggui Fang

**Affiliations:** 1 College of Horticulture, Nanjing Agricultural University, Nanjing, 210095 China; 2 College of Agro-grassland Science, Nanjing Agricultural University, Nanjing, 210095 China

**Keywords:** Grape berry, Berry shape, Tomato analyzer, SNP, Candidate gene

## Abstract

**Background:**

In the breeding of new horticultural crops, fruit shape is an important selection characteristic. A variety of fruit shapes appeared during the gradual process of selection and domestication. However, few studies have been conducted on grape berry shape, especially studies related to mining candidate genes. To discover candidate genes related to grape berry shape, the present study first took the berry shape parameters analyzed by Tomato Analyzer as the target traits and used a genome-wide association analysis to analyze candidate genes.

**Results:**

In total, 122 single-nucleotide polymorphism (SNP) loci had significant correlations with multiple berry shape traits in both years, and some candidate genes were further mined. These genes were mainly related to LRR receptor-like serine/threonine-protein kinase (At1g05700 and At1g07650), transcription factors (GATA transcription factor 23-like, transcription factor VIP1, transcription initiation factor TFIID, and MADS-box transcription factor 6), ubiquitin ligases (F-box protein SKIP19 and RING finger protein 44), and plant hormones (indole-3-acetic acid-amido synthetase GH3.6 and ethylene-responsive transcription factor ERF061). In addition, some important SNP loci were associated with multiple berry-shape traits. The study further revealed some genes that control multiple traits simultaneously, indicating that these berry shape traits are subject to the coordinated regulation of some genes in controlling berry shape.

**Conclusions:**

In the present work, we identified interesting genetic determinants of grape berry shape-related traits. The identification of molecular markers that are closely related to these berry-shape traits is of great significance for breeding specific berry-shaped grape varieties.

**Supplementary Information:**

The online version contains supplementary material available at 10.1186/s12870-022-03434-x.

## Background

Fruit is the main product of fleshy horticultural plants. In the process of breeding new varieties, fruit size and shape are important selection characteristics [1]. During gradual selection and domestication, varieties with different fruit sizes and shapes have appeared [1, 2]. In terms of fruit size, cultivated plants usually have larger fruits than wild varieties. In terms of fruit shape, wild fruits are usually round, but cultivated plants have fruits of various shapes [3]. Fruit shape is an important criterion in the development of new varieties to meet specific market needs [4].

In the breeding of new varieties, the fruit processing industry, and the fresh market industry, fruit shape is an important descriptive character that cannot be ignored. The importance of fruit shape is reflected in the registration of new plant varieties and new descriptions of existing varieties, mechanized fruit picking, consumer preference assessments [5–7], genetic trait surveys [8], and fruit transportation [6]. The shape of the tomato fruit determines its culinary use (fresh, sliced, diced, processed, or cooked) and its market value [5]. Flat tomato fruits are popular in homes and restaurants, whereas slender and slightly blocky fruits are easier to harvest and machine process than round fruits and therefore are favored by the processing industry [3].

The classification of fruit shape is the premise of studying the genetic mechanism of fruit shape in horticultural crops. Generally, tomato varieties are correctly classified according to the fruit morphology described by the International Union for the Protection of New Plant Varieties (UPOV) and the International Plant Genetic Resources Institute (IPGRI) [9, 10]. According to the UPOV and IPGRI classification standards for tomato varieties, based on the analysis of fruit shape using analysis software, the tomato variety shapes are divided into eight categories: flat, round, heart-shaped, bull heart shape, long, rectangular, obovate and ellipse [5, 11]. In addition, the shapes of other fruits (including cherry, eggplant and watermelon) have also been classified [12–14]. However, few reports have been provided the classification of grape berry shape.

Given the importance of fruits with different shapes, researchers have conducted extensive research on fruit shapes, and a series of advances have been made [5]. These studies have mainly included morphology and genetics [15–17]. From the perspective of morphology, related studies have shown that mature fruit morphology is highly correlated with the ovary, and fruit morphology can be determined before ovary pollination [16, 17]. That is to say, the structure and morphology of the fruit are determined during flower development [15]. From the perspective of genetics, fruit shape is a complex trait controlled by multiple genes through different pathways [18]. Several genes controlling tomato fruit shape have been cloned [15, 19]. *SUN* and *OVATE* control elongated shapes, and both *FASCIATED* (*FAS*) and *LOCULE NUMBER* (*LC*) alter locule number, which has an impact on shape [2]. The allelic distribution of *SUN*, *OVATE*, *LC*, and *FAS* genes is closely related to UPOV and IPGRI fruit classification [2].

*SUN* encodes a protein that is a member of the IQ67-domain (IQD) protein families, and that is a positive regulator of growth, leading to elongated fruit [20]. The mutation of *SUN* is the result of a gene replication event mediated by the retrotransposon *Rider* [21]. Overexpression of *SUN* results in very elongated parthenocarpic fruits in addition to twisted stems and leaf axes [22]. Further study found that, *SUN* changed the expression of auxin-related genes, including those involved in auxin biosynthesis, homeostasis, signal transduction, and polar transport, indicating that *SUN* may regulate the ovary/fruit shape by regulating the expression of auxin-related genes in the early stage of ovary formation [23]. In addition, studies have shown that *SUN* has no significant effect on fruit weight, and it regulates tomato fruit shape by changing the cell division mode (increasing longitudinal cell division and reducing transverse fruit cell division) and re-regulating fruit quality [22]. *OVATE* encodes a negative regulator of growth, which may be an inhibitor of transcription, thus reducing the length of fruit [19, 24]. The fruit regulated by the *OVATE* allele carries an early termination codon; this allele is presumed to be an invalid allele [23]. A mutation in *FAS* resulted in flattened tomatoes due to an increase in the number of ventricles that affect fruit quality [25]. Further studies have shown that the underlying gene of *FAS* is *CLAVATA3* (*CLV3*) [26], and the down-regulation of this gene is caused by large insertion in the first intron (estimated to be 6–8 kb), resulting in fruits with high locule numbers [25]. In addition to tomato [19–25], more and more genes related to fruit shape have been revealed in other horticultural crops, such as watermelon [27], peach [28–31] and cucumber [32–34].

With the rapid development of molecular biology, the genetic mechanism of fruit shape has gradually been revealed [35]. Through map-based cloning, protein interaction studies, and genome editing, a common genetic mechanism for morphological diversity in fruit and other plant organs has been identified [35]. Namely, the cell division pattern during ovary development is regulated by the OVATE Family Protein (OFP) and TONNEAU1 Recruiting Motif (TRM) proteins, thereby changing the final fruit shape [35]. Furthermore, research suggests that OFPs and TRMs control the shapes of fruits, tubers, vegetables and grains in domesticated plants, and that the apparent universality of this OFP-TRM module may be part of the network required for coordinated multicellular growth in all plants [35].

However, compared with other horticultural crops, few studies have examined grape berry shape, and berry shape-related gene mining has not been reported. Grape (*Vitis vinifera* L.) is one of the most widely cultivated fruit crops [36]. Grape berries are commercially grown for fresh fruits, juices and raisins, but are used mainly for fermentation into wine [36]. Berry development is a complex process that involves profound physiological and metabolic changes [37]. At the stage of berry ripening, further metabolic changes make the fruit edible and attractive, which promotes the spread of seeds, including changes in peel color, cell swelling and an influx of water, the softening of berries, the accumulation of sugar in the pulp, the loss of organic acids and tannic acid and volatile aroma synthesis [37]. Grapes are favored by consumers because of their high nutritional value. With the improvement of people’s living standards, some traditional berry varieties no longer meet the needs of consumers. People have begun to pursue novel, special, high-quality berries. Cultivating and selling varieties with peculiar fruit shapes can greatly improve economic benefits. As an important appearance quality, fruit shape has drawn increasing attention from consumers and producers.

Similar to tomatoes, wild grape germplasm resources are generally round in shape, black-purple in color, and smaller in size. With constant selection, berries of various types have been produced. At present, the mining of fruit shape genes mainly uses quantitative trait loci (QTL) mapping [18, 38], and less research on fruit shape-related genes has used genome-wide association analysis. Therefore, in this study, we first used Tomato Fruit Analyzer to analyze grape berry shape-related trait parameters and then used a genome-wide association study to analyze berry shape-related genes in order to reveal the molecular mechanism of different berry shapes and to provide theoretical references for cultivating target berry shapes. Studies have shown that fruit shape is determined at an early stage [39]. Therefore, the genes related to cell division and expansion may also play an important role in determining the shape of grape berries. However, the specific genes need to be identified and studied.

## Methods

### Plant materials and sample collection

A total of 279 grape varieties were used in the present study. These varieties were collected from different countries, including Afghanistan, Albania, Armenia, Azerbaijan, Brazil, Bulgaria, Canada, China, France, Georgia, Greece, Hungary, Israel, Italy, Japan, Lebanon, Moldavia, Moldova, Romania, Russia, South Africa, Spain, Tajikistan, Turkey, the United Kingdom, the United States of America and Uzbekistan. These varieties were cultivated at the Zhengzhou Fruit Research Institute, Chinese Academy of Agricultural Sciences (113°39′'E, 34°43′'N). We included 205 *V. vinifera* L. specimens and 74 *V. vinifera* × *V. labrusca* specimens, as shown in Table S1. All of the materials were collected and preserved by Nanjing Agricultural University, China (118°78′'E, 31°51′'N). All necessary permits for planting and investigating the natural population were obtained from Nanjing Agricultural University, China. The planting direction was oriented north-south; the vines were pruned into two branches, with one or two clusters per branch; and branches were pruned vertically to about 1.5 m. Conventional pest management but no growth regulators were used during plant growth. Unless otherwise stated, we sampled berries between 08:00 and 10:00 in the morning. Berries with the same level of maturity and no defects on the berry surface were selected for testing.

### Experimental methods

#### Classification of the grape berry shape

Based on the UPOV (UPOV, 2001) and IPGRI (IPGRI, 1996) classification systems, we divided the berry shapes of the varieties in the present study into nine different berry shapes: flat round, heart-shaped, curve-shaped, obovoid, ovoid, elliptic, round, long elliptic and long round.

#### Analysis of grape berry shape-related parameters using the tomato analyzer

We selected five berries with essentially the same size at maturity, cut them longitudinally with a surgical blade, and photographed the samples with reference to [11]. We used the Tomato Analyzer 3.0 (The Ohio State University, Columbus, OH, USA) to determine the following indicators: perimeter, area, width mid-height, maximum width, height mid-width, maximum height, curved height, fruit shape index external I, fruit shape index external II, curved fruit shape index, proximal fruit blockiness, distal fruit blockiness, fruit shape triangle, shoulder height, proximal angle micro, proximal angle macro, proximal indentation area, distal angle micro, distal angle macro, width widest pos (the ratio of the height at which the maximum width occurs to the maximum height), eccentricity, proximal eccentricity, distal eccentricity, fruit shape index internal and eccentricity area index [40].

#### Whole-genome resequencing and reference genome information

The DNA of 279 grape varieties was extracted using a plant genome DNA kit (Tiangen Biotech (Beijing) Co. Ltd., Beijing, China), and the DNA of qualified samples was sequenced with an Illumina HiSeqTM 2500 (Illumina, Inc., CA, USA) [41]. The average sequencing depth of each material was expected to be 8 × for the development of single nucleotide polymorphism (SNP) markers within the population. We used the grape genome (PN40024) as the reference genome. The grape genome (PN40024) was downloaded from: ftp://ftp.ensemblgenomes.org/pub/release-23/plants/fasta/vitis_vini-fera/dna/ [42].

#### Identification of SNP markers

Sequencing reads were compared to the reference genome by BWA software (Wellcome Trust Sanger Institute, Hinxton, Cambridge, UK) [43], and the genome-wide SNP markers were developed by GATK software (Ohio Supercomputer Center, Columbus, OH, USA) [44] and SAMtools software (Wellcome Trust Sanger Institute, Hinxton, Cambridge, UK) [45]. High-quality SNP markers were filtered for downstream analysis. The following steps were used to filter: (1) minor allele frequency (MAF) > 0.05 and (2) call rate > 50%, from which highly consistent population SNPs were obtained.

#### Population structure and attenuation analysis of linkage disequilibrium at the population level

The population structure of the 279 samples was analyzed using ADMIXTURE software (University of California, Los Angeles, CA, USA) [46] with the following operating parameters: the number of subgroups (K-value) ranging from 2 to 20, K of iterative operations starting from 2 and the number of runs and repetitions of each time set to 10,000. According to the K-value with the lowest error rate in the cross validation, the optimal number of subgroups was determined. PopLDdecay software (Xi’an Jiaotong University, Xi’an, China) [47] was used to analyze the Linkage Disequilibrium (LD) at the population level, and the parameters were set at -MAF 0.05 -MaxDist 500 -Miss 0.25.

#### GWAS

In the process of GWAS analysis, individual genetic relationships and population structure are the main factors resulting in false-positive associations. The GWAS was based on SNPs and used TASSEL software (Cornell University, Ithaca, NY, USA) [48] to obtain correlation values using a compressed mixed linear model (MLM). The formula is as follows: Y = αX + βQ + μK + e, α, β, μ, and e. In the equation, Y is the phenotypic trait, X is the indicator matrix of the genotype (fixed effect), α is the estimated parameter of fixed effect, Q is the indicator matrix of population genetic structure, β is the effect of SNP, K is the indicator matrix of the individual genetic relationship, μ is the predicted random individual, and e is the random residual, obeying e ~ (0, δ_e_^2^). Among them, the sample population structure Q (Fig. S1) was calculated by ADMIXTURE software (University of California, Los Angeles, CA, USA) [46], and the affinity K between samples was calculated using SPAGeDi software (Université Libre de Bruxelles, Brussels, Belgium) [49]. MLMs use Q + K information. Finally, each SNP locus can obtain a correlation value (*P*). The *P*-values were corrected using Bonferroni’s method: α ≤ 0.1 and α ≤ 0.05 (*P* ≤ 1.77 × 10^− 7^ and *P* ≤ 8.33 × 10^− 8^, respectively) [50].

#### Annotation of genes related to grape berry-shape traits

Based on the 566,129 SNPs developed from 279 grape varieties (Table S2), the LD of SNPs in all samples was analyzed, denoted by r^2^ (Fig. S2). The r^2^ value decays to half of the initial value of 6.15 kb. A 6-kb region was taken from the upstream and downstream of the SNP sites with associations, and functional genes for the associated regions were mined. We used the Clusters of Orthologous Genes (COG), Gene Ontology (GO), Kyoto Encyclopedia of Genes and Genomes (KEGG), Swiss-Prot and Non-redundant (NR) databases for gene annotation according to the regions formed by the associated SNPs.

#### Expression analysis of candidate genes for grape berry-shape traits

The expression values of the candidate genes for fruit shape traits in the pericarp (including the skin and flesh), flower, and seed were screened from the Gene Expression Omnibus (GEO Datasets, No.GSE36128) [51]. The logarithm of the original value based on 10 was taken, and the heat map was drawn with Excel 13 (Microsoft Corporation, Redmond, Washington, USA).

### Statistical analysis

Due to the high correlation of most fruit shape traits in 2 years, we used the mean value of two-year data for Principal Component Analysis (PCA), correlation analysis, and variation analysis. PCA and variation analysis were carried out using SPSS version 16.0 (IBM, Armonk, NY, USA). Linear correlation analysis was performed using Excel 13 (Microsoft Corporation, Redmond, Washington, USA).

## Results

### Quantitative distribution of different berry shapes in grapes

**Fig. 1:**
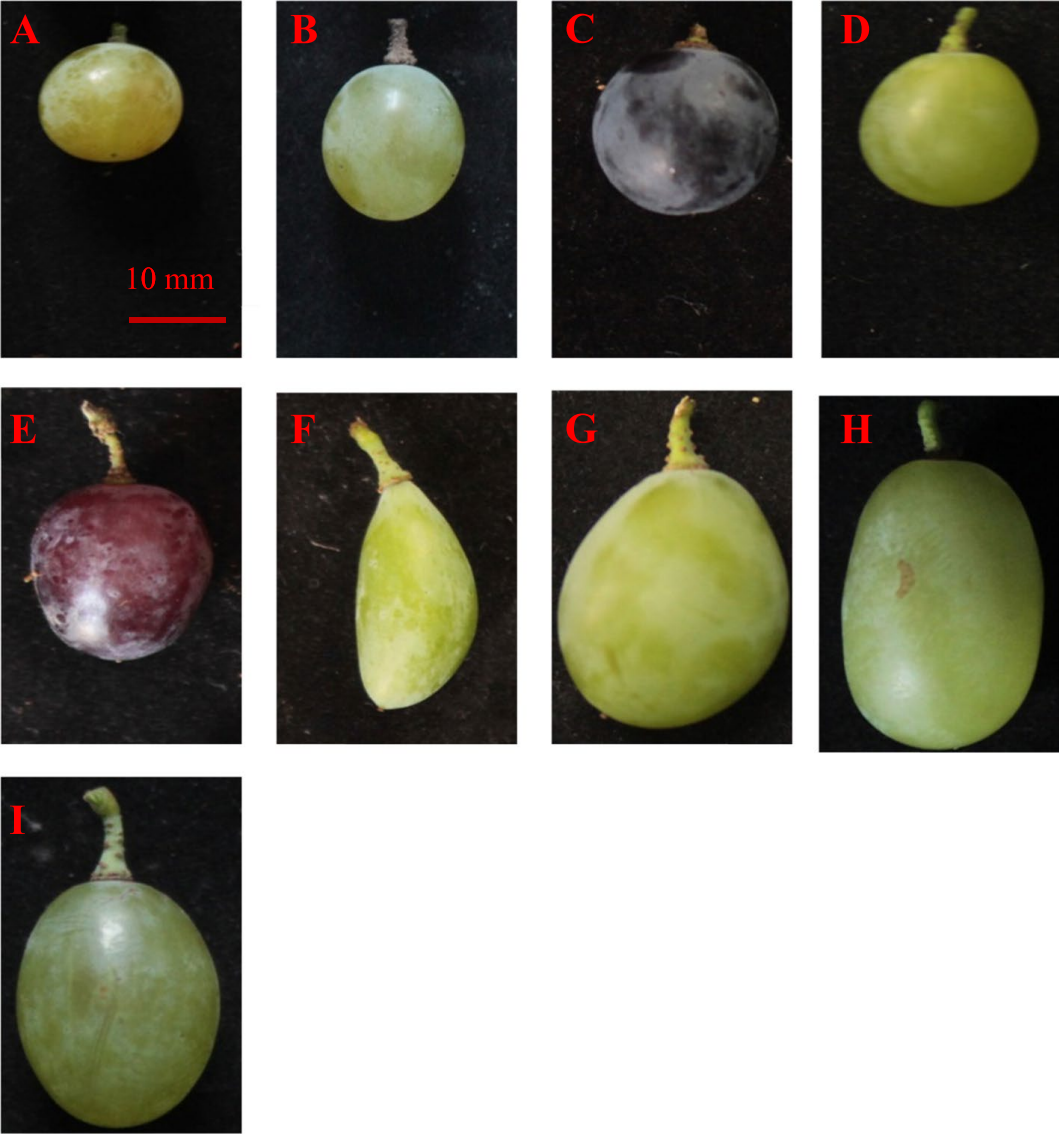
Representative grape varieties with different shapes. **A**: flat round, Yiliang; **B**: elliptical, Lady Washington; **C**: round, Lival; **D**: heart-shaped, Kamea; **E**: ovoid, Jingkejing; **F**: curve-shaped, Lünai; **G**: obovoid, Beni Fuji; **H**: long elliptical, Qichakapulie; **I**: long round, Manai

**Fig. 2:**
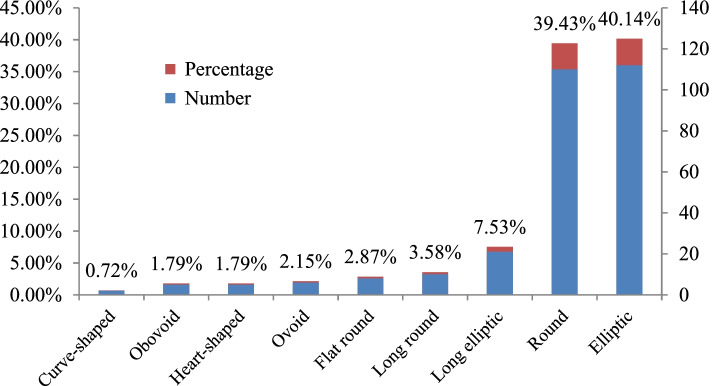
Quantitative distribution of different berry-shapes in grape varieties

In the present study, there were nine different fruit shapes: flat round, heart-shaped, obovoid, ovoid, curve-shaped, elliptic, round, long elliptic and long round (some representative varieties are shown in Fig. 1). As shown in Fig. 2, the number distribution of varieties with elliptic shape was the largest at 112, accounting for 40.14% of the varieties, followed by the round berry shape at 110, accounting for 39.43% of the varieties. The varieties with long elliptic or long round berry shapes were relatively fewer, with 21 and 10 varieties, respectively, accounting for 7.53 and 3.58% of the varieties, respectively. The curve-shaped varieties were the least common, with only two, accounting for 0.72% of the varieties. In addition, the number of the varieties with flat round; heart-shaped, ovoid and obovoid berry shapes was between 5 and 8, accounting for 1.79–2.87% of the investigated varieties.

### Distribution of different grape berry-shape morphologies and quantities of the *V. vinifera* L. and *V. vinifera* × *V. labrusca*

The number distribution of different grape berry shapes of the *V. vinifera* L. and *V. vinifera* × *V. labrusca* is shown in Table 1. From the perspective of berry shape types, there were nine different berry shapes in the *V. vinifera* L.: curve-shaped, flat round, heart-shaped, obovoid, ovoid, elliptic, round, long elliptic and long round. There were five different berry shapes in the *V. vinifera* × *V. labrusca*: curve-shaped, flat round, obovoid, elliptic, and round. From the distribution of the four different berry shapes common in the *V. vinifera* L. and *V. vinifera* × *V. labrusca*, 62.5% of flat round shapes were distributed in the *V. vinifera* L.; 80% of obovoid berries were distributed in the *V. vinifera* × *V. labrusca*; and 20% obovoid berries were distributed in the *V. vinifera* L. The distributions of elliptic berries in the *V. vinifera* L. and *V. vinifera* × *V. labrusca* were 71.43 and 28.57%, respectively; 68.18% of round berries were distributed in *V. vinifera* L., and 31.82% were distributed in *V. vinifera* × *V. labrusca*.

**Table 1:** The distribution of different berry shapes in the *V. vinifera* L. and *V. vinifera* × *V. labrusca*

Grape berry shape	Distribution of different berry shapes in the *V. vinifera* L. and *V. vinifera* × *V. labrusca*	
	*V. vinifera* L.	*V. vinifera* × *V. labrusca*
Curve-shaped	1 (50.00%)	1 (50.00%)
Obovoid	1 (20.00%)	4 (80.00%)
Heart-shaped	5 (100.00%)	0 (0.00%)
Ovoid	6 (100.00%)	0 (0.00%)
Flat round	5 (62.50%)	3 (37.50%)
Long round	10 (100.00%)	0 (0.00%)
Long elliptic	21 (100.00%)	0 (0.00%)
Round	75 (68.18%)	35 (31.82%)
Elliptic	80 (71.43%)	32 (28.57%)

The number outside the brackets indicates the number of varieties in the interval, and the number inside the brackets indicates the percentage of the varieties in the interval

### Variation of different grape berry-shape parameters using the tomato analyzer

**Fig. 3:**
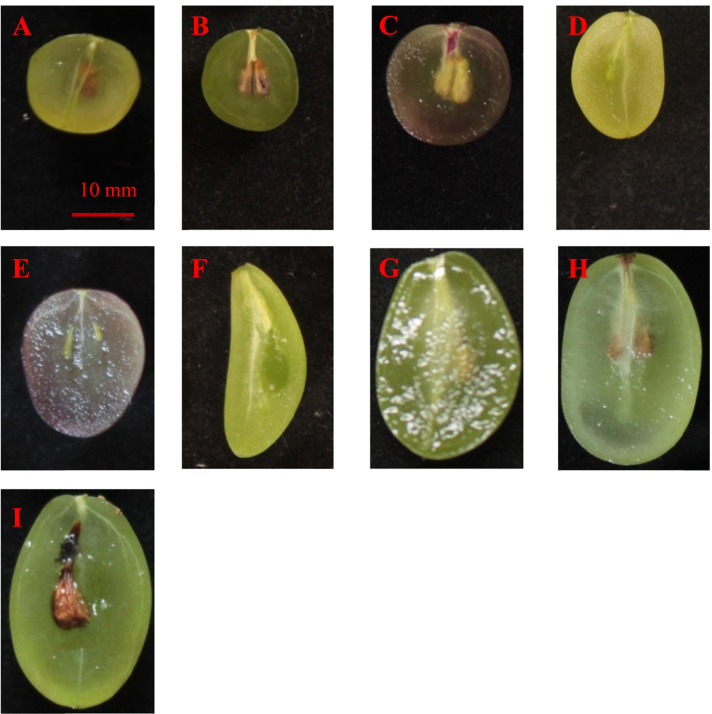
Longitudinal section of the representative grape varieties with different shapes. **A**: flat round, Yiliang; **B**: elliptical, Lady Washington; **C**: round, Lival; **D**: heart-shaped, Kamea; **E**: ovoid, Jingkejing; **F**: curve-shaped, Lünai; **G**: obovoid, Beni Fuji; **H**: long elliptical, Qichakapulie; **I**: long round, Manai

**Fig. 4:**
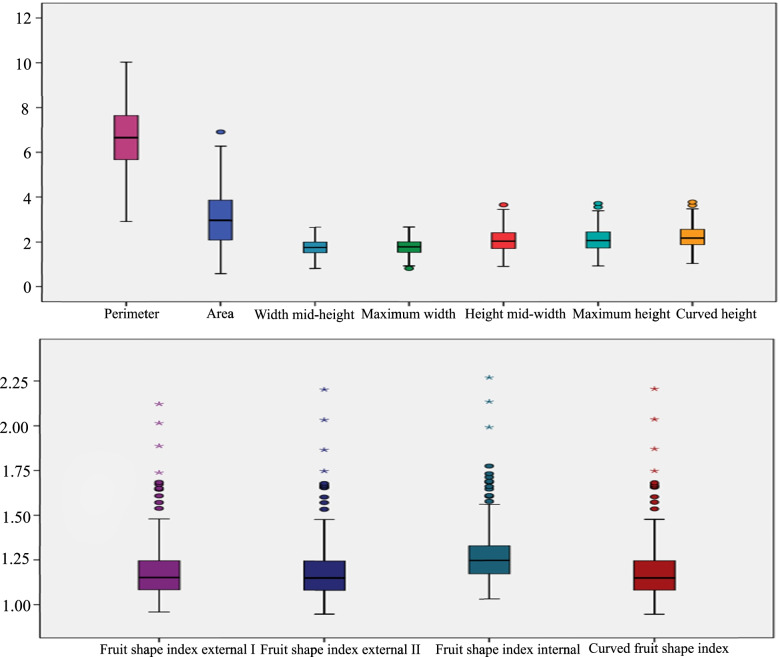
The distribution of the basic measured characters and shape index parameters of grape berries

The longitudinal section of representative grape varieties of different shapes is shown in Fig. 3. Box diagrams of the grape berry shape-related parameters are shown in Fig. 4 and Fig. S3; these include the variation of these berry shape parameters. Table S3 shows that the variation in different berry shape parameters was different, and the variation range was 0.18% (distal eccentricity) to 63.64% (proximal indentation area). We found that among these parameters, the traits with a coefficient of variation less than 1% included distal eccentricity and proximal eccentricity; traits with a coefficient of variation higher than 20% included the maximum width, width mid-height, perimeter, curved height, maximum height, height mid-width, area, shoulder height and proximal indentation area.

### The PCA and correlation analysis of grape berry shape-related parameters

**Fig. 5:**
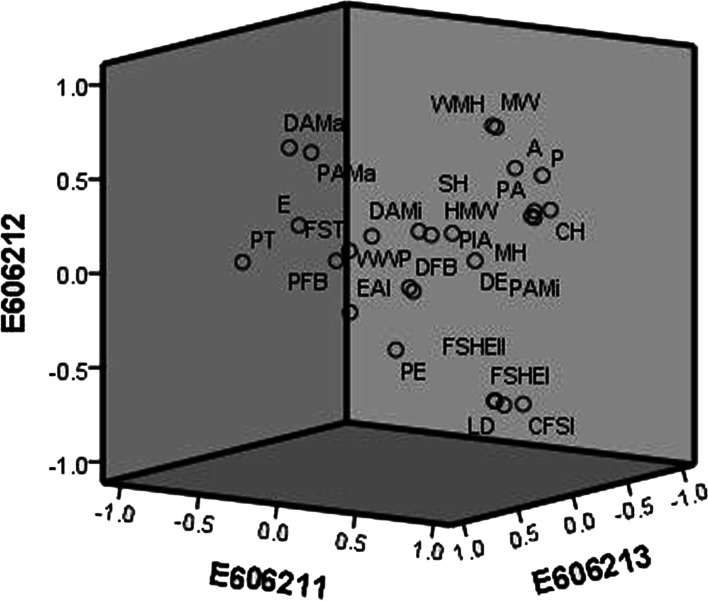
Principal component analysis (PCA) of grape berry shape-related traits. E606211: PCA1; E606212: PCA2; E606213: PCA3. P: perimeter; A: area; WMH: width mid-height; MW: maximum width; HMW: height mid-width; MH: maximum height; CH: curved height; FSIEI: fruit shape index external I; FSIEII: fruit shape index external II; CFSI: curved fruit shape index; FSII: fruit shape index internal; PFB: proximal fruit blockiness; DFB: distal fruit blockiness; EST: fruit shape triangle; E: eccentricity; PE: proximal eccentricity; DE: distal eccentricity; WWP: width widest pos; EAI: eccentricity area index; PAMi: proximal angle micro; PAMa: proximal angle macro; DAMi: distal angle micro; DAMa: distal angle macro; PIA: proximal indentation area; SH: shoulder height

**Fig. 6:**
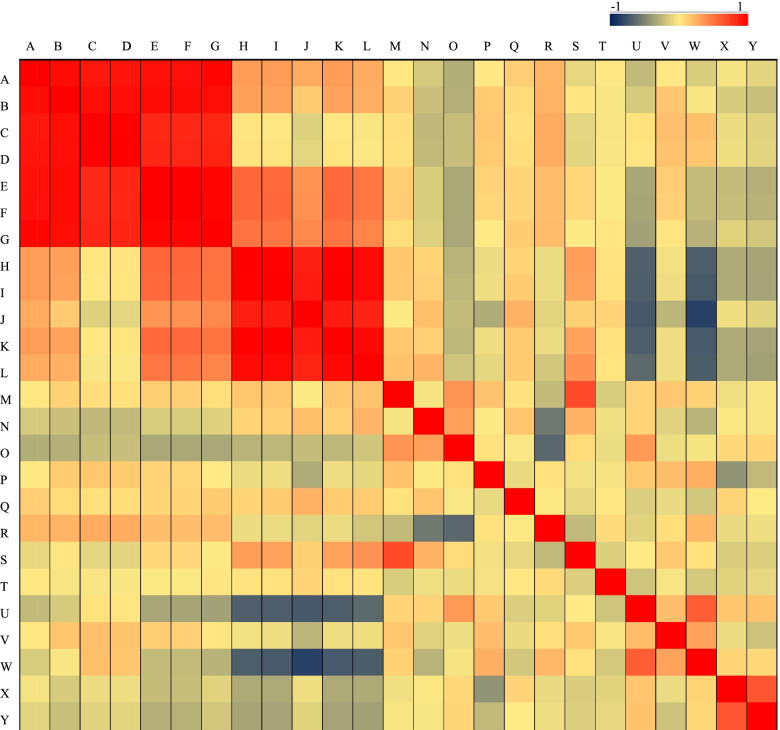
Correlation analysis of grape berry shape-related parameters. **A**: perimeter; **B**: area; **C**: width mid-height; **D**: maximum width; **E**: height mid-width; **F**: maximum height; **G**: curved height; **H**: fruit shape index external I; **I**: fruit shape index external II; **J**: fruit shape index internal; **K**: curved fruit shape index; **L**: proximal fruit blockiness; **M**: distal fruit blockiness; **N**: fruit shape triangle; **O**: eccentricity; **P**: proximal Eccentricity; **Q**: distal eccentricity; **R**: width widest pos; **S**: eccentricity area index; **T**: proximal angle micro; **U**: proximal angle macro; **V**: distal angle micro; **W**: distal angle macro; **X**: proximal indentation area; **Y**: shoulder height

The PCA of grape berry shape-related parameters is shown in Fig. 5, Table S4, and Table S5. As shown in Table S4, the characteristic values of the first five components in this study were all greater than 1, and the cumulative contribution rate was 78.426%, indicating that the explanatory rate of these five factors to the whole population was nearly 80%, so the first five factors could be extracted. The cumulative contribution rate of the first two principal components reached 54.752%. The perimeter, area, width mid-height, maximum width, height mid-width, maximum height, curved height, fruit shape index external I, fruit shape index external II, curved fruit shape index, and fruit shape index internal were highly correlated with PCA1 and PCA2. These characters contained most of the variation of fruit shape characters. The correlation analysis of the same fruit shape-related parameters in 2 years (2019 and 2020) is shown in Fig. S4. For most fruit shape traits, the correlation of two-year data is high, which indicates that these traits have high heritability. In addition, the correlation analysis of the grape berry shape-related parameters is shown in Fig. 6 and Table S6. The seven traits of perimeter, area, width mid-height; maximum width, height mid-width, maximum height and curved height were positively correlated with each other, and the coefficients were 0.836–0.999. The fruit shape index external I, fruit shape index external II, fruit shape index internal and curved fruit shape index were positively correlated with each other, and the correlation coefficients were 0.854–0.997.

### Results of grape berry shape traits in the GWAS

**Fig. 7:**
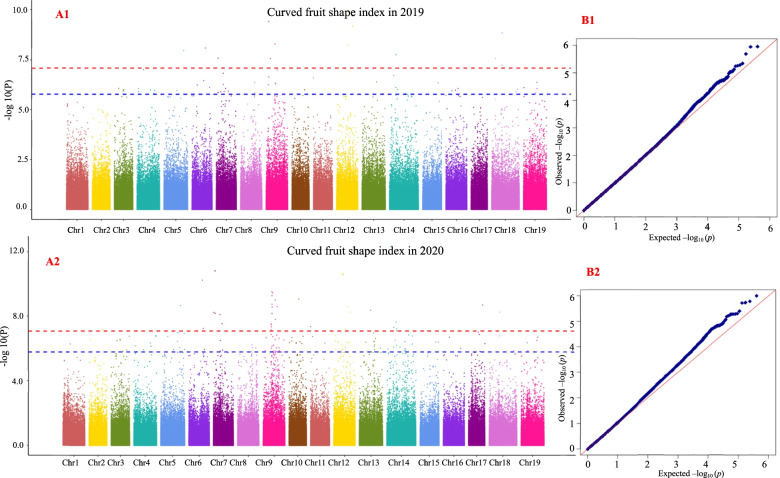
Genome-wide association study with the MLM for curved fruit shape index. A1 and A2 are Manhattan plots of the mixed linear model (MLM). The abscissa represents the position of the chromosome, and the ordinate represents the *P*-value (−log10 P). The negative logarithm of the base 10 and the scattered points (or lines) on the graph represent the −log10 (p) corresponding to each SNP locus. The red dotted line is the negative logarithm of 0.05/all SNPs, and the blue dotted line is the negative logarithm of 0.1/all SNPs. Scattered dots (or lines) above the threshold line are candidate sites. B1 and B2 are QQ-plot plots. The abscissa represents the expected value, and the ordinate represents the observed value. In the initial stage, the actual observed *P*-value was close to the expected *P*-value, indicating that the influence of population structure on the association analysis could be effectively controlled under this model, and thus false positives could also be effectively controlled. The same applies below

**Fig. 8:**
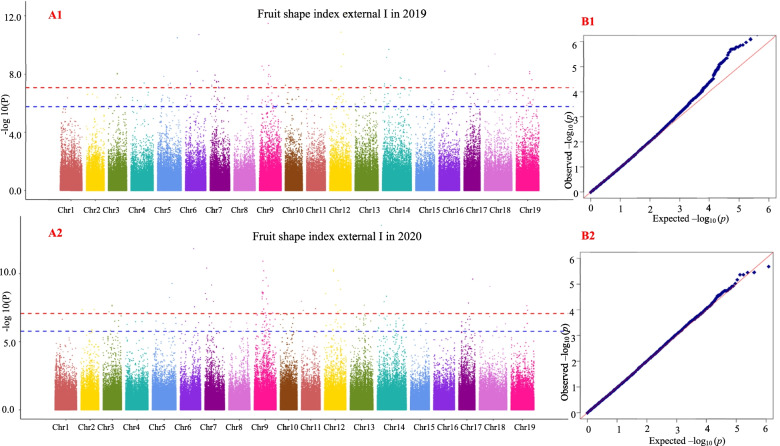
Genome-wide association study with the MLM for fruit shape index external I

**Fig. 9:**
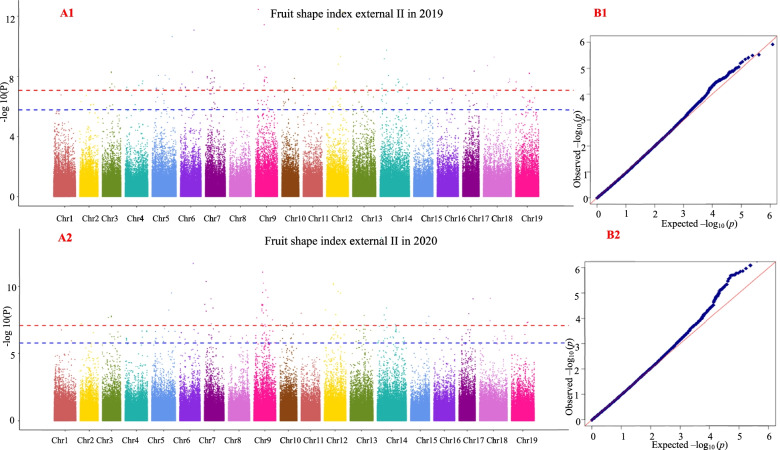
Genome-wide association study with the MLM for fruit shape index external II

**Fig. 10:**
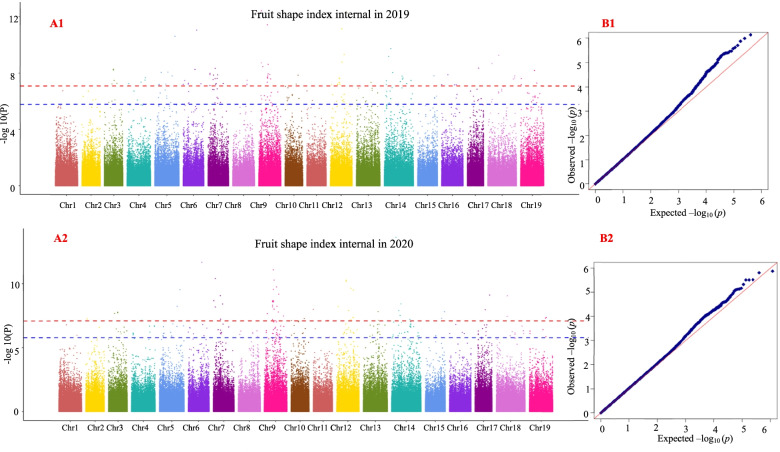
Genome-wide association study with the MLM for fruit shape index internal

Using the 25 fruit shape traits in 2019 and 2020 as the target traits, GWAS was performed using MLM. The GWAS results showed that the four fruit shape traits analyzed in this study (curved fruit shape index, fruit shape index external I, fruit shape index external II and fruit shape index internal) were significantly correlated with multiple SNP loci within 2 years. Therefore, this study only provides the analysis results of these four traits. GWAS results with the MLM for curved fruit shape index, fruit shape index external I, fruit shape index external II, and fruit shape index internal are shown in Figs. 7, 8, 9 and 10, respectively, and the detailed results are shown in Table S7, Table S8, Table S9, and Table S10, respectively.

### SNP loci associated with simultaneous control of multiple fruit shape traits

As shown in Table S11, we found that multiple berry shape traits were associated with the same SNP loci and the same berry shape trait was significantly associated with multiple SNP loci. In both years, 122 SNP loci had significant correlations with multiple berry shape traits, which were distributed on 19 chromosomes. Among them, 18 SNPs were located on chromosome 9, 16 SNPs were distributed on chromosome 12 and one SNP was distributed on chromosomes 1, 2, 11 and 15. The number of SNPs distributed on other chromosomes ranged from 3 to 16.

The grape berry shape traits of curved fruit shape index, fruit shape index external II, and fruit shape index internal were associated with four significant SNP loci (marker names: 17_173150, 17_414350, 17_43912 and 17_584380) in 2 years; fruit shape index external I and fruit shape index external II were associated with one significant SNP locus (marker name: 17_604843) in 2 years; curved fruit shape index, fruit shape index external I, fruit shape index external II and fruit shape index internal were associated with 29 significant SNP loci (marker name: 17_103635, 17_158117, 17_165943, 17_165944, 17_165946, 17_165948, 17_165952, 17_173305, 17_176706, 17_211324, 17_230957, 17_304846, 17_305195, 17_309493, 17_312547, 17_326935, 17_335450, 17_337013, 17_337015, 17_482077, 17_491897, 17_516416, 17_528926, 17_552659, 17_572735, 17_590009, 17_88457, 17_88457 and 17_88459) in 2 years.

A1 and A2 are Manhattan plots of the mixed linear model (MLM). The abscissa represents the position of the chromosome, and the ordinate represents the *P*-value (−log10 P). The negative logarithm of the base 10 and the scattered points (or lines) on the graph represent the −log10 (p) corresponding to each SNP locus. The red dotted line is the negative logarithm of 0.05/all SNPs, and the blue dotted line is the negative logarithm of 0.1/all SNPs. Scattered dots (or lines) above the threshold line are candidate sites. B1 and B2 are QQ-plot plots. The abscissa represents the expected value, and the ordinate represents the observed value. In the initial stage, the actual observed *P*-value was close to the expected *P*-value, indicating that the influence of population structure on the association analysis could be effectively controlled under this model, and thus false positives could also be effectively controlled. The same applies below.

### Polygenic control of grape berry shape traits

In our analysis, we found that some candidate genes were candidates for multiple berry shape traits, and the same trait could be coordinated by multiple genes (Table 2). The relevant candidate genes unearthed in this study mainly included genes related to transcription factors, cell wall metabolism, plant hormones, ubiquitin ligases and serine/threonine protein kinases. Five transcription factor-related genes (*VIT_02s0025g01360*, *VIT_06s0004g01280*, *VIT_12s0057g00880*, *VIT_16s0022g-02330* and *VIT_18s0076g00330*), four cell wall metabolism-related genes (*VIT_07s0005g04110*, *VIT_08s0007g00440*, *VIT_08s0007g00290* and *VIT_09s0096g00850*), two plant hormones-related genes (*VIT_02s0025g01360* and *VIT_12s0134g00230*), and two LRR receptor-like serine/threonine protein kinase genes (*VIT_09s0002g03030* and *VIT_09s0070g00140*) were associated with the fruit shape index external II and fruit shape index internal; two ubiquitin ligases-related genes (*VIT_03s0088g01090* and *VIT_10s0003g04300*) were associated with the fruit shape index external I, fruit shape index external II and fruit shape index internal. The results showed that these berry shape traits were co-regulated by some genes involved in the regulation of berry morphology.

**Table 2:** The candidate genes of significantly associated regions for multiple berry shape traits

Berry shape traits	Gene ID	Location	Nr annotation
Fruit Shape Index External II, Fruit Shape Index Internal	*VIT_03s0097g00140*	3:9892772–9,893,537	PREDICTED: *Vitis vinifera* L-type lectin-domain containing receptor kinase IX.1 (LOC100265547), mRNA
	*VIT_04s0023g01970*	4:18530909–18,534,706	PREDICTED: *Vitis vinifera* putative glucose-6-phosphate 1-epimerase (LOC100266694), mRNA
	*VIT_06s0004g00020*	6:95994–97,288	PREDICTED: *Vitis vinifera* NAC domain-containing protein 68 (LOC100263939), mRNA
	*VIT_06s0004g01280*	6:1524152–1,537,550	PREDICTED: GATA transcription factor 23-like [*Vitis vinifera*]
	*VIT_07s0005g04080*	7:7175304–7,176,037	PREDICTED: *Vitis vinifera* MDIS1-interacting receptor like kinase 2-like (LOC100246300), mRNA
	*VIT_07s0005g04100*	7:7199256–7,202,858	PREDICTED: *Vitis vinifera* MDIS1-interacting receptor like kinase 2-like (LOC100266867), mRNA
	*VIT_08s0007g00290*	8:14603038–14,609,600	PREDICTED: *Vitis vinifera* pectin acetylesterase 5 (LOC100244164), transcript variant X1, mRNA
	*VIT_08s0007g00440*	8:14732840–14,734,936	PREDICTED: *Vitis vinifera* expansin-A6 (LOC100245911), mRNA
	*VIT_09s0002g03020*	9:2728009–2,740,198	PREDICTED: *Vitis vinifera* putative leucine-rich repeat receptor-like protein kinase At2g19210 (LOC100266874), mRNA
	*VIT_09s0002g03030*	9:2778212–2,782,247	PREDICTED: *Vitis vinifera* probable LRR receptor-like serine/threonine-protein kinase At1g05700 (LOC100251452), mRNA
	*VIT_09s0070g00850*	9:14657091–14,663,881	PREDICTED: *Vitis vinifera* probable LRR receptor-like serine/threonine-protein kinase At1g07650 (LOC104878156), transcript variant X1, mRNA
	*VIT_10s0092g00200*	10:11506152–11,510,357	PREDICTED: GDP-mannose transporter GONST3 [*Vitis vinifera*]
	*VIT_12s0134g00230*	12:7753336–7,755,364	PREDICTED: *Vitis vinifera* indole-3-acetic acid-amido synthetase GH3.6 (LOC100255170), mRNA
	*VIT_12s0134g00240*	12:7768803–7,769,369	PREDICTED: *Vitis vinifera* calcium-binding protein PBP1 (LOC100265313), mRNA
	*VIT_16s0022g02330*	16:14940190–14,955,349	PREDICTED: *Vitis vinifera* MADS-box transcription factor 6 (LOC100256085), transcript variant X1, mRNA
	*VIT_19s0014g00550*	19:560043–564,043	PREDICTED: *Vitis vinifera* TOM1-like protein 2 (LOC100264036), transcript variant X1, mRNA
	*VIT_19s0014g00560*	19:572717–579,014	PREDICTED: *Vitis vinifera* probable arabinosyltransferase ARAD1 (LOC100241759), transcript variant X2, mRNA
Curved Fruit Shape Index, Fruit Shape Index External II, Fruit Shape Index Internal	*VIT_07s0005g04110*	7:7204959–7,209,235	PREDICTED: *Vitis vinifera* cellulose synthase A catalytic subunit 4 [UDP-forming] (LOC100241197), mRNA
Fruit Shape Index External I, Fruit Shape Index External II, Fruit Shape Index Internal	*VIT_02s0025g01360*	2:1274105–1,275,209	PREDICTED: ethylene-responsive transcription factor ERF061 [*Vitis vinifera*]
	*VIT_07s0005g02370*	7:4729295–4,730,035	PREDICTED: *Vitis vinifera* germin-like protein 5–1 (LOC100260641), mRNA
	*VIT_07s0005g02380*	7:4743365–4,744,223	PREDICTED: *Vitis vinifera* germin-like protein subfamily 2 member 4 (LOC100267594), mRNA
	*VIT_09s0002g08430*	9:9424676–9,434,529	PREDICTED: *Vitis vinifera* protein NUCLEAR FUSION DEFECTIVE 4 (LOC100262975), mRNA
	*VIT_09s0096g00850*	9:12518294–12,520,356	PREDICTED: *Vitis vinifera* probable polygalacturonase At3g15720 (LOC100251699), mRNA
	*VIT_09s0070g00140*	9:13115563–13,117,457	PREDICTED: *Vitis vinifera* CBL-interacting serine/threonine-protein kinase 5 (LOC100255067), mRNA
	*VIT_09s0018g02060*	9:19788292–19,790,440	PREDICTED: *Vitis vinifera* sugar transport protein 8 (HT14), mRNA
	*VIT_10s0003g04300*	10:7394269–7,397,548	PREDICTED: *Vitis vinifera* F-box protein SKIP19 (LOC100265193), transcript variant X1, mRNA
	*VIT_10s0116g00680*	10:300617–304,777	PREDICTED: *Vitis vinifera* homeobox-leucine zipper protein MERISTEM L1 (LOC100264009), mRNA
	*VIT_12s0057g00880*	12:9587450–9,598,955	PREDICTED: transcription initiation factor TFIID subunit 15b [*Vitis vinifera*]
	*VIT_13s0019g05160*	13:6937392–6,997,802	PREDICTED: *Vitis vinifera* pullulanase 1, chloroplastic (LOC100247866), transcript variant X1, mRNA
	*VIT_18s0076g00330*	18:16209694–16,218,061	PREDICTED: *Vitis vinifera* transcription factor VIP1 (LOC100241011), mRNA
Curved Fruit Shape Index, Fruit Shape Index External I, Fruit Shape Index External II, Fruit Shape Index Internal	*VIT_16s0013g00860*	16:6368533–6,436,142	PREDICTED: *Vitis vinifera* polycomb group protein EMBRYONIC FLOWER 2 (LOC100252876), transcript variant X1, mRNA
	*VIT_09s0002g08370*	9:9246440–9,256,248	PREDICTED: *Vitis vinifera* protein TOPLESS (LOC100248092), mRNA
	*VIT_13s0064g00360*	13:21879764–21,898,935	PREDICTED: *Vitis vinifera* notchless protein homolog (LOC100262007), transcript variant X1, mRNA
	*VIT_03s0088g01090*	3:9340156–9,341,562	PREDICTED: *Vitis vinifera* RING finger protein 44 (LOC104878839), mRNA

### Tissue expression analysis of candidate genes annotated by grape fruit shape traits

**Fig. 11:**
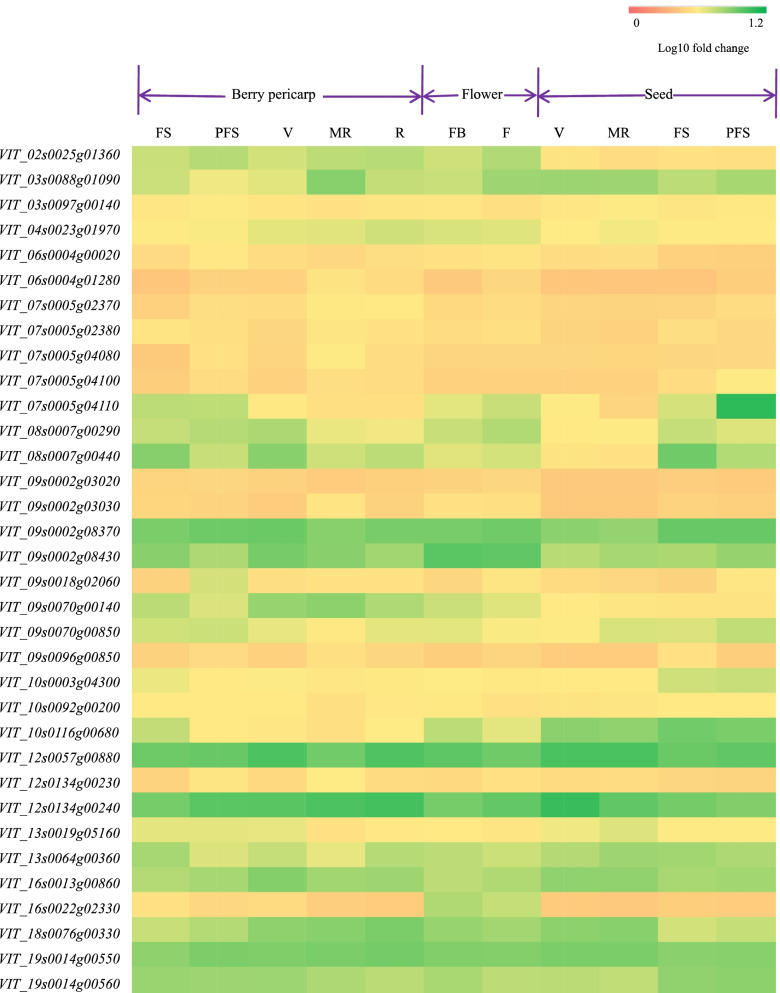
Tissue expression analysis of candidate genes annotated by grape fruit shape traits. FS: fruit set; PFS: post-fruit set; V: veraison; MR: mid-ripening; R: ripening; FB: flowering begins (10% caps off); F: flowering (50% caps off)

Tissue expression analysis of candidate genes annotated by grape fruit shape traits was performed by GEO Datasets (No.GSE36128) [51], as shown in Fig. 11. The results showed that two plant hormone-related genes (*VIT_02s0025g01360*, ethylene-responsive transcription factor ERF061; *VIT_12s0134g00230*, incole-3-acetic acid-amido synthetase GH3.6), two ubiquitin ligase-related genes (*VIT_03s0088g0109*, RING finger protein 44; *VIT_10s0003g04300*, F-box protein SKIP19), two LRR receptor-like serine/ threonine-protein kinase-related genes (*VIT_09s0002g03030*, At1g05700; *VIT_09-s0070g00850*, At1g07650); four cell wall metabolism-related genes (*VIT_07s0005g04110*, cellulose synthase A catalytic subunit 4; *VIT_08s0007g0029*0, pectin acetylesterase 5; *VIT_08s0007g00440*, expansin-A6; *VIT_09s0096g00850*, probable polygalacturonase At3g15720), and other genes were expressed to varying degrees in grape pericarp, flowers, and seeds at different developmental stages. Thus, these genes can be used as candidate genes for grape fruit shape traits.

## Discussion

Compared with wild varieties with round fruits, a cultivar’s fruit shape has a high degree of diversity [2]. To meet different market demands, various types of fruit shapes have gradually been produced during the genetic improvement of horticultural crops [1–3]. Compared with the fruit shape research in tomato [3, 6], watermelon [17] and sweet pepper [18], few studies have examined grape berry shapes. The mining of berry shape-related genes holds great significance for breeding new grape varieties with different fruit shapes.

Measuring the fruit morphology and color characteristics of vegetable and fruit crops in an objective and reproducible manner is important for the detailed phenotypic analysis of these traits [40]. The Tomato Analyzer is a software program that measures 37 two-dimensional shape-related attributes in a semi-automated and reproducible manner [11, 52].

### Analysis of the variability of grape fruit shape-related traits using the tomato analyzer

The output produced by the Tomato Analyzer can be used in many applications. In genetic research, the output has been used to detect fruit shape QTLs in several isolated populations derived from crosses between different cultivated tomato varieties, including LA1589 (*Solanum lycopersicum*) and wild species *Solanum pimpinellifolium* accessions [11, 53]. The Tomato Analyzer also has been used for shape diversity [54] and fruit color [55] studies. In this study, we used the Tomato Analyzer to analyze 25 tomato shape-related traits in 279 varieties. We found that these fruit shape-related variations ranged from 0.18 to 63.64%. Among them, the distal eccentricity and proximal eccentricity had relatively small variation, while proximal indentation area and shoulder height exhibited relatively large variation. For other fruit-shaped traits, the degree of variation was between 2.00 and 40.24%. Some studies have examined the genetic variation of fruit traits using the Tomato Analyzer. A previous study found that the broad heritability of most fruit traits was high in tomato and *Capsicum annuum* [56, 57]. The broad heritability of shoulder height was 0.56 [56]. In the present study, the coefficient of variation of shoulder height was found to be high, which indicated that it responded to variety characteristics.

For the studies on candidate genes related to fruit shape, in addition to tomatoes [2, 20, 25, 51], in-depth research has been conducted in horticultural crops such as peaches [28–31] and cucumbers [32, 33]. However, few studies have examined candidate genes related to grape shape.

### GWAS of genes related to grape berry shape traits

In this study, we used a genome-wide association study to analyze grape berry shape as the target trait using the Tomato Analyzer and mined some of the candidate genes that control grape berry shape. The relevant candidate genes unearthed in this study included genes related to plant hormones, ubiquitin ligases, LRR receptor-like serine/threonine-protein kinase, and transcription factors.

### GWAS of plant hormone-related genes related to grape berry shape characters

In the present study, through the mining of functional gene-associated regions, we identified two genes related to plant hormones that were related to berry shape—namely, *VIT_12s0134g00230* (indole-3-acetic acid-amido synthetase GH3.6) and *VIT_02s0025g01360* (ethylene-responsive transcription factor ERF061). These two genes were identified as candidate genes for the berry shape traits fruit shape index external II and fruit shape index internal.

Plant hormones play an important role in fruit organ morphogenesis and development [58–61]. Studies have identified auxin and gibberellin as early signs of fruit setting and fruit growth [58–60]. Both hormones have a positive effect on cell division and cell expansion [61]. *AtOFP1* is reported to be located in the nucleus and acts as an active transcriptional repressor, regulating a gene in the gibberellin biosynthetic pathway (*AtGA20ox1*). The reduction of cell elongation is partly caused by the inhibition of gibberellin biosynthesis [24]. Further research showed that *CaOvate* down-regulated the *CaGA20ox1* gene, which was similar to the tomato *GA20ox1* gene. *CaGA20ox1* regulates the effect of *CaOvate* on fruit elongation [62]. Unfortunately, the gibberellin-related gene was not linked in this study. In addition, a previous study pointed out that calcium signaling regulates cell polarity and cell elongation by regulating auxin transport in tobacco [63]. The number and size of short fruit cells were lower than those of long fruit, which may have been caused by abnormal auxin signal transduction in short fruit [64]. Studies have shown that the binding of indole-3-acetic acid (IAA)-amido synthetases with amino acids is an important aspect of auxin stability in vivo [65, 66]. In addition to auxin and gibberellin, ethylene content may also be involved in fruit morphogenesis [58]. Studies have suggested that genes involved in hormone action, such as ethylene-related genes, are upregulated in fruits, indicating that genes related to cell cycle control and hormone action may contribute to the process of fruit development from cell division to cell expansion [58]. Based on gene chips from GEO Datasets (No.GSE36128) [51], we analyzed the expression levels of these two plant hormone-related genes (*VIT_12s0134g00230* and *VIT_02s0025g01360*) in pericarp, flower, and seed, and found that the two hormone-related genes were expressed to varying degrees in different grape organs at different growth and development stages, indicating that these two genes may be involved in the formation of grape fruit organ morphology. On the basis of findings from these related studies, supported by the results of this study, the two genes *VIT_12s0134g00230* (indole-3-acetic acid-amido synthetase GH3.6) and *VIT_02s0025g01360* (ethylene-responsive transcription factor ERF061) may affect grape berry morphology by regulating auxin and ethylene content, but the specific mechanism requires further study.

### GWAS of ubiquitin ligase-related genes related to grape berry-shape characters

In the present study, through the mining of functional gene-associated regions, we identified two genes related to ubiquitin ligases that were related to berry shape—namely, one RING finger protein 44 -related gene (*VIT_03s0088g01090*) and one F-box protein SKIP19-related gene (*VIT_10s0003g04300*). The two genes are candidate genes for the berry characteristics fruit shape index external I, fruit shape index external II and fruit shape index internal. In addition, the gene *VIT_ 03s0088g01090* is also a candidate gene of the curved fruit shape index. Ubiquitination is a fine post-translational modification that is widely found in all eukaryotes [67]. Ubiquitin is a conserved protein with 76 amino acids that has a high degree of conservation and involves various aspects of cell physiology [67, 68].

RING-type E3 is one of the ubiquitin ligases, and many studies have been conducted on its regulation of plant organ morphology [67, 68], especially the regulation of seed organs [67]. Seed size is an important agronomic trait. Several regulatory pathways that determine seed size have been identified, among which RING-type E3 ligases are involved, mainly by regulating gametogenesis and cell cycle processes. RING-type E3 DA2 negatively regulates seed size by reducing cell proliferation and synergistic interactions with the ubiquitin receptor DA1 in developing seeds. The ubiquitin receptor DA1 is also a key regulator of seed size [69]. Previous studies have shown that the DA2 homolog RING-type E3 OsGW2 (Grain Width and Weight 2) in rice has a negative effect on particle size and final yield by mediating cell division [70]. In addition to controlling seed shape, ubiquitin ligase may also play an important role in controlling fruit shape. In the present study, the two ubiquitin ligase genes *(VIT_03s0088g01090* and *VIT_10s0003g04300*) associated with multiple berry shape traits were normally expressed in the pericarp (as shown in Fig. 10), suggesting that they may play an important role in regulating berry morphogenesis. The in-depth mechanism of the regulation of grape berry shape traits by two ubiquitin ligase genes (*VIT_03s0088g01090* and *VIT_10s0003g04300*) needs further study.

### GWAS association analysis of LRR receptor-like serine/threonine-protein kinase genes related to grape berry-shape traits

In the present study, the functional gene mining of berry-shaped trait-associated regions was performed, and two LRR receptor-like serine/threonine-protein kinase genes (*VIT_09s0002g03030,* At1g05700; *VIT_09s0070g00850,* At1g07650) were obtained. Both genes are located on chromosome 9 and are candidate genes for fruit shape index external II and fruit shape index internal. Some research has suggested that the shape of the pit in peach can be used to distinguish traditional varieties [71]. Mapping-based cloning methods have revealed that candidate genes for this trait may be LRR-RLK protein kinases rather than *MADS-box* genes [72]. Although tissue expression patterns (as shown in Fig. 10) showed that two LRR receptor-like serine/threonine-protein kinase genes (*VIT_09s0002g03030*, At1g05700; *VIT_09s0070g00850*, At1g07650) were normally expressed in grape pericarp, the expression of the two genes in different fruit shapes was not analyzed. The specific mechanism of *VIT_05s0020g03030* and *VIT_09s0070g00850* regulating the traits fruit shape index external II and fruit shape index internal needs further study.

In addition, we examined some transcription factors (transcription factor VIP1, GATA transcription factor 23-like, transcription initiation factor TFIID and MADS-box transcription factor 6) related to grape berry shape in this study. Tissue expression patterns showed that these transcription factor correlations were expressed to a certain extent in tissues at different stages of grape development (Fig. 10). However, few reports, are available about the relationship between these genes and fruit shape. Whether these genes regulate grape berry shape and the specific mechanism needs to be further studied.

## Conclusion

To discover candidate genes related to grape berry shape, the present study first took fruit shape parameters analyzed by the Tomato Analyzer as the target traits and used genome-wide association study to analyze candidate shape related genes. The relevant candidate genes unearthed in this study included genes related to plant hormones, ubiquitin ligase, LRR receptor-like serine/threonine-protein kinase and transcription factors. The present study increased the understanding of the genetic control of grape berry shape traits. The identification of molecular markers that are closely related to these berry shape traits holds great significance for breeding specific berry shape varieties.

## Supplementary Information

**Additional file 1: Figure S1.** Population structure of natural populations.

**Additional file 2: Figure S2.** Attenuation analysis of LD at the population level.

**Additional file 3: Figure S3.** Distribution of the other morphological traits of grape berries.

**Additional file 4: Figure S4.** Correlation analysis of the same berry shape-related traits across 2 years.

**Additional file 5: Table S1.** Experimental material used in this study.

**Additional file 6: Table S2.** SNP number distribution on each chromosome.

**Additional file 7: Table S3.** The variation in different berry shape parameters.

**Additional file 8: **T**able S4.** Total variance explained.

**Additional file 9: Table S5.** Component matrix of grape berry shape-related parameters.

**Additional file 10: Table S6.** Correlation analysis of grape berry shape-related parameters.

**Additional file 11: Table S7.** Details of SNP loci associated with curved fruit shape index identified via GWAS from both years.

**Additional file 12: Table S8.** Details of SNP loci associated with fruit shape index external I identified via GWAS from both years.

**Additional file 13: Table S9.** Details of SNP loci associated with fruit shape index external II identified via GWAS from both years.

**Additional file 14: Table S10.** Details of SNP loci associated with fruit shape index internal identified via GWAS from both years.

**Additional file 15: Table S11.** Details of SNP loci associated with multiple berry-shape traits identified via GWAS from both years.

## Data Availability

The datasets generated during and/or analysed during the current study are included in its supplementary information files. The raw Illumina sequencing data from this study have been submitted to NCBI Sequence Read Archive (SRA) under the accession number PRJNA782678.

## Abbreviations

BWA: Burrow-Wheeler Aligner
CLAVATA3: CLV3
COG: Clusters of Orthologous Genes
DNA: Deoxyribonucleic acid
FAS: FASCIATED
GA: Gibberellic acid
GEO: Gene Expression Omnibus
GO: Gene Ontology
GW2: Width and Weight 2
GWAS: Genome-wide association study
IAA: Indole 3-acetic acid
IPGRI: International Plant Genetic Resources Institute
IQD: IQ67-domain
KEGG: Kyoto Encyclopedia of Genes and Genomes
LC: LOCULE NUMBER
LD: Linkage Disequilibrium
LRR: Leucine-rich repeat
MAF: Minor allele frequency
MLM: Mixed linear model
NR: Non-redundant
OFP: OVATE Family Protein
PCA: Principal Component Analysis
QTL: Quantitative trait loci
Ring: Really interesting gene
SNP: Single-nucleotide polymorphism
TRM: TONNEAU1 Recruiting Motif
UPOV: International Union for the Protection of New Varieties of Plants
