# Supplementary material for: Genome-wide association study of the candidate genes for grape berry shape-related traits
Source: BMC Plant Biol. 2022 Jan 20;22:42. doi: 10.1186/s12870-022-03434-x (PMC8772106; doi:10.1186/s12870-022-03434-x)
Supplement: Supplementary file 1 — Additional file 1: Figure S1. Population structure of natural populations. [file 12870_2022_3434_MOESM1_ESM.docx]

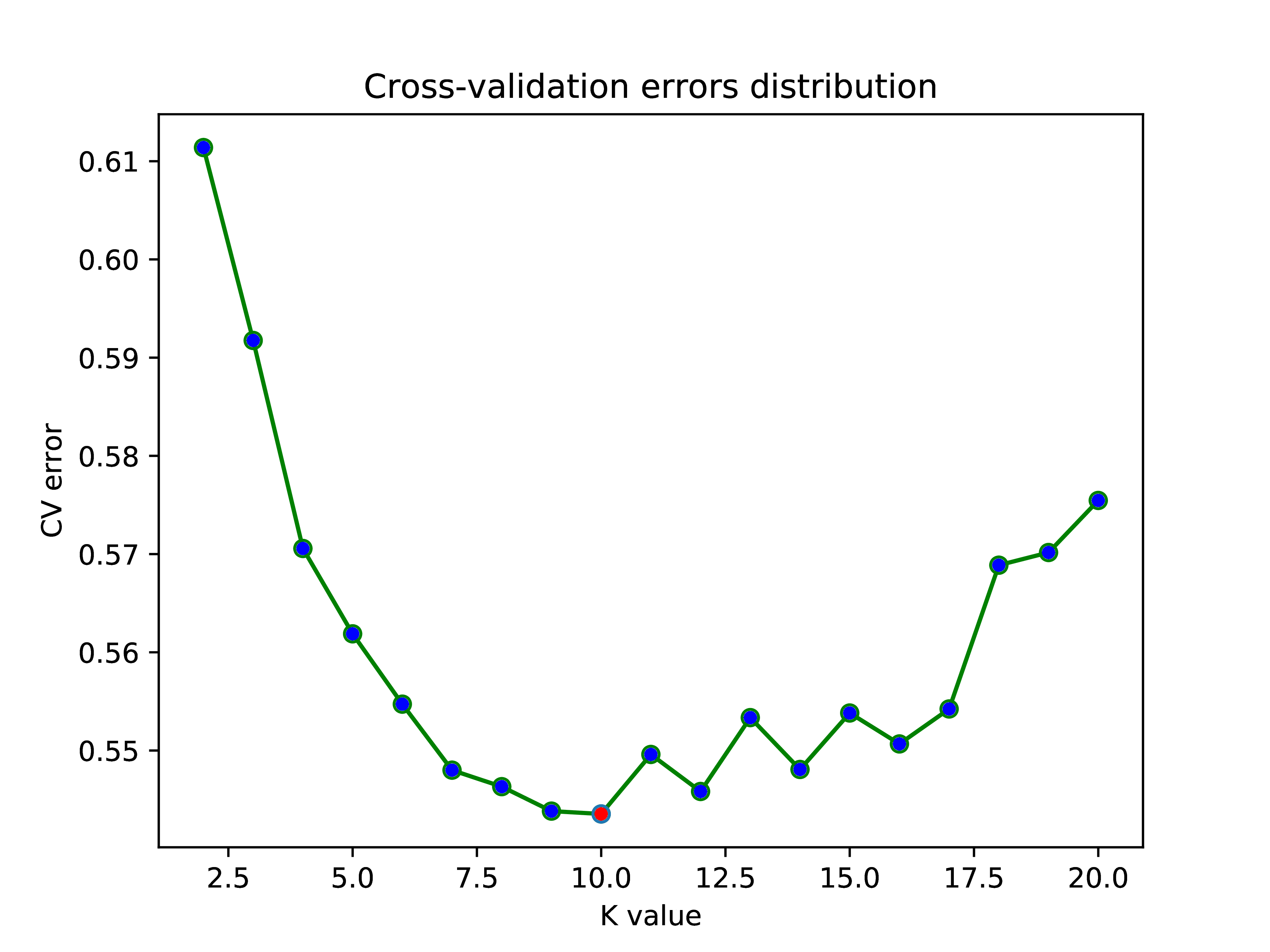


**B**


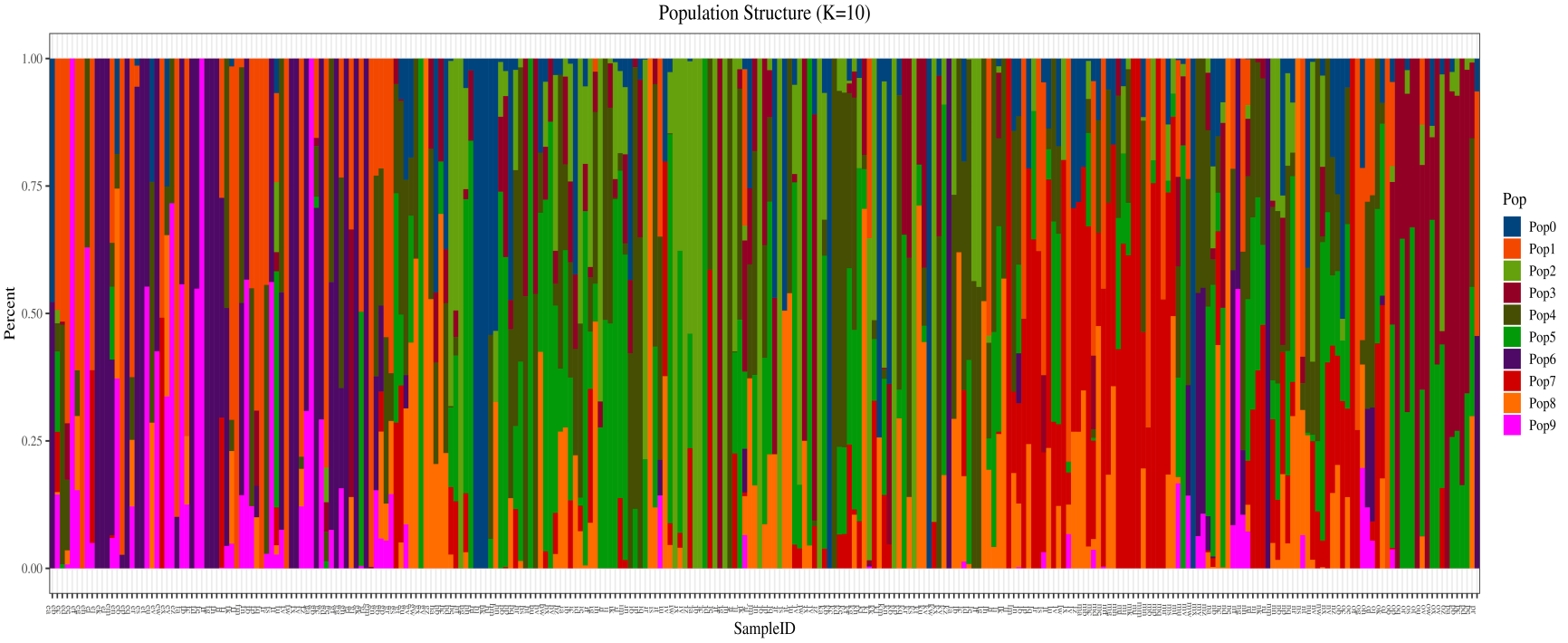


**A**

K=10

Fig. S1 Population structure of natural populations

A: The group structure of 279 grape varieties. The abscissa is the structure of each sample, and each column represents an individual, and the length of different colored fragments represents the proportion of an ancestor in the genome of an individual. K=2 to 20 on the left side of the figure indicates that the number of assumed ancestor groups ranges from 2 to 20, and the K-value is the number of subgroups or ancestors contained in the sample. B: The cross validation (CV) value corresponding to each K-value was extracted, and the K-value corresponding to the minimum CV value is the optimal subgroup grouping number
