# Supplementary figures and images for: Genome-wide association study of the candidate genes for grape berry shape-related traits
Source: BMC Plant Biol. 2022 Jan 20;22:42. doi: 10.1186/s12870-022-03434-x (PMC8772106; doi:10.1186/s12870-022-03434-x)

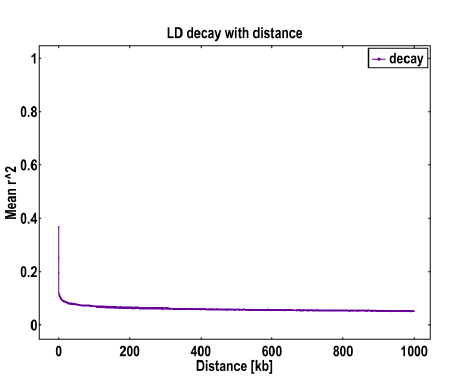


Fig. S2 Attenuation analysis of LD at the population level

Supplement: Supplementary file 2 — Additional file 2: Figure S2. Attenuation analysis of LD at the population level. [file 12870_2022_3434_MOESM2_ESM.docx]
