# Supplementary material for: Genome-wide association study of the candidate genes for grape berry shape-related traits
Source: BMC Plant Biol. 2022 Jan 20;22:42. doi: 10.1186/s12870-022-03434-x (PMC8772106; doi:10.1186/s12870-022-03434-x)
Supplement: Supplementary file 3 — Additional file 3: Figure S3. Distribution of the other morphological traits of grape berries. [file 12870_2022_3434_MOESM3_ESM.docx]

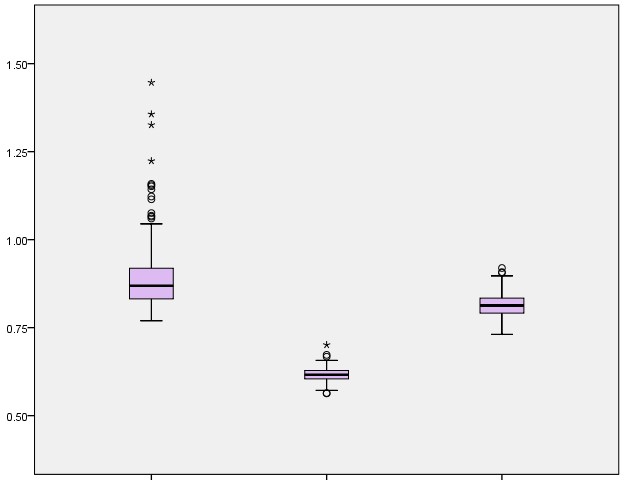


Proximal fruit blockiness

Distal fruit blockiness

Fruit shape triangle


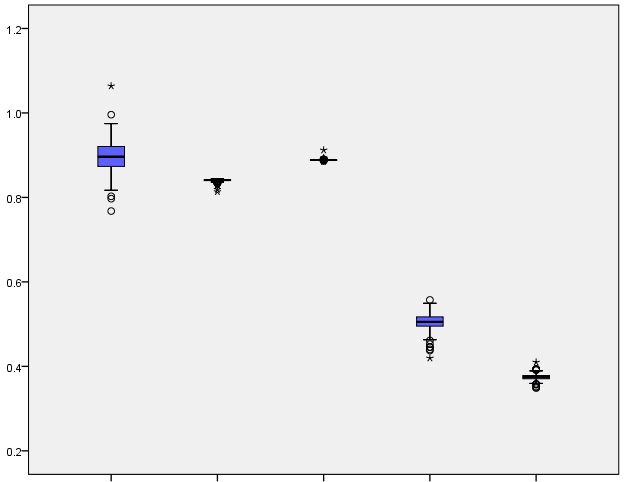


Eccentricity

Proximal eccentricity

Distal eccentricity

Width widest pos

Eccentricity area index


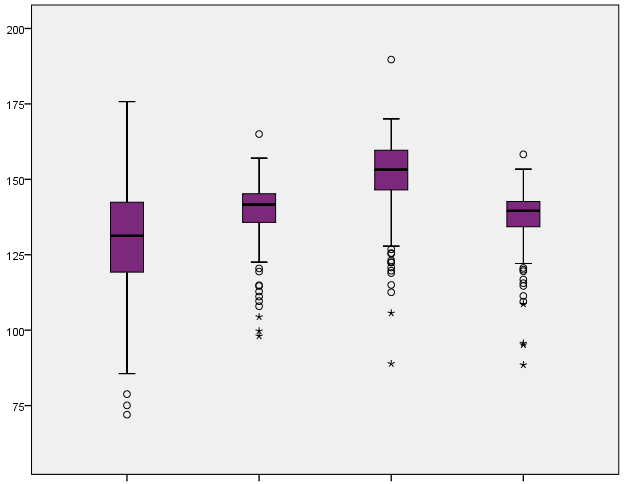


Proximal angle micro

Proximal angle macro

Distal angle micro

Distal angle macro


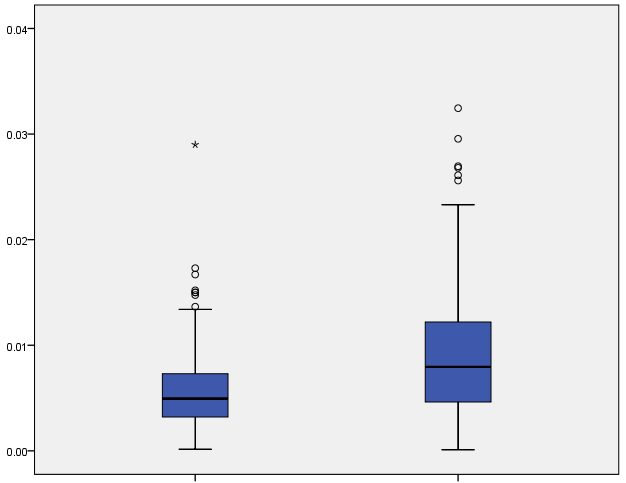


Proximal indentation area

Shoulder height

Fig. S3 Distribution of the other morphological traits of grape berries
