# Supplementary material for: Genome-wide association study of the candidate genes for grape berry shape-related traits
Source: BMC Plant Biol. 2022 Jan 20;22:42. doi: 10.1186/s12870-022-03434-x (PMC8772106; doi:10.1186/s12870-022-03434-x)
Supplement: Supplementary file 4 — Additional file 4: Figure S4. Correlation analysis of the same berry shape-related traits across 2 years. [file 12870_2022_3434_MOESM4_ESM.docx]

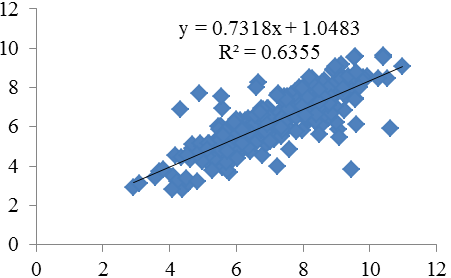

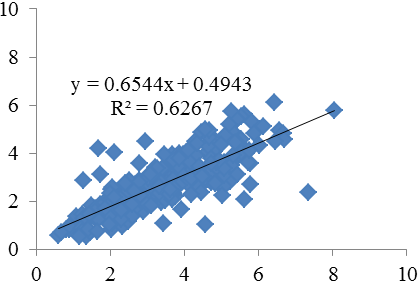

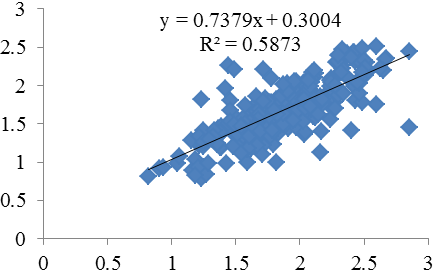

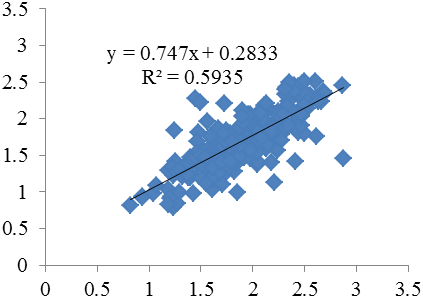

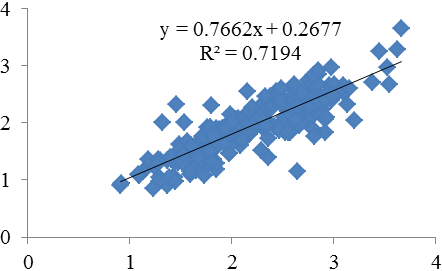

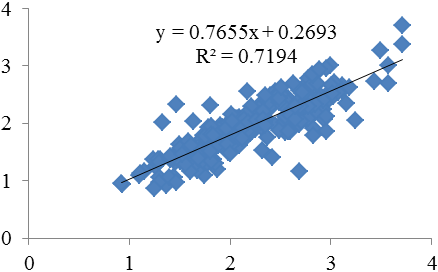

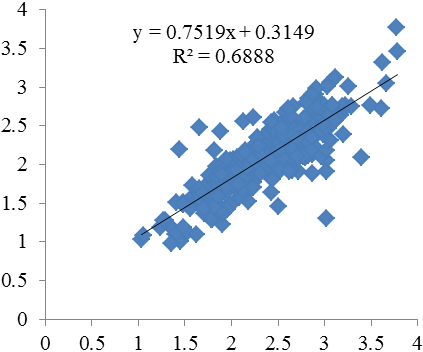

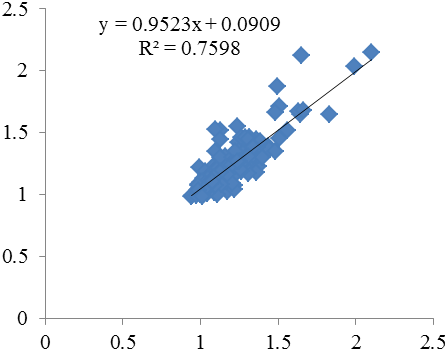


Perimeter in 2019

Perimeter in 2020

Area in 2019

Area in 2020

Width mid-height in 2019

Width mid-height in 2020

Maximum width in 2019

Maximum width in 2020

Height mid-width in 2019

Height mid-width in 2020

Maximum height in 2019

Maximum height in 2020

Curved height in 2019

Curved height in 2019

Fruit shape index external I in 2019

Fruit shape index external I in 2020


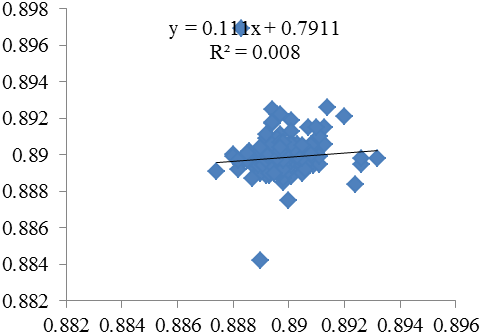

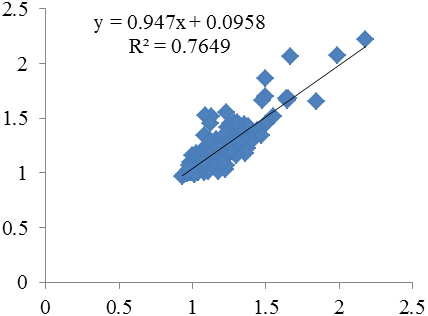

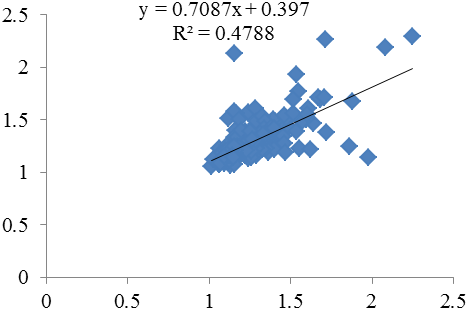

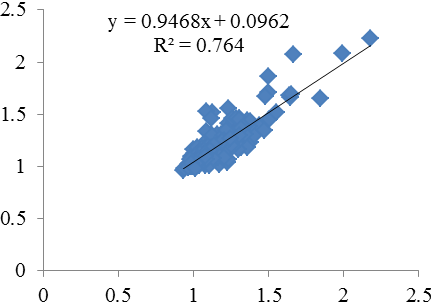

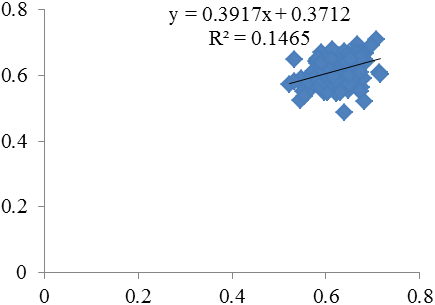

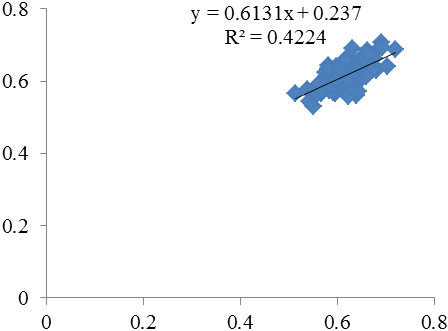

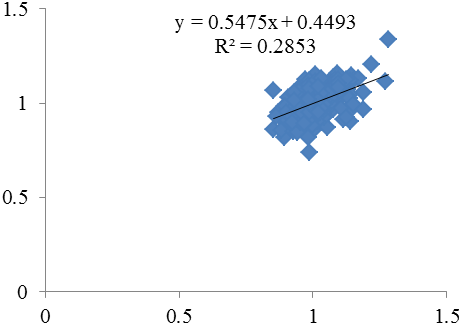

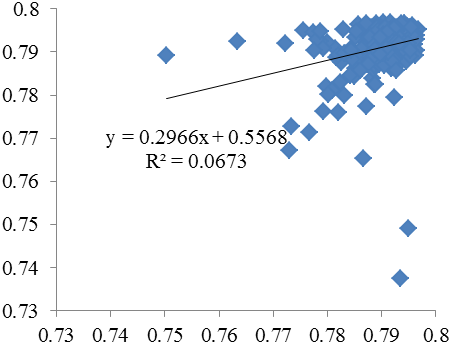


Fruit shape index external II in 2019

Fruit shape index external II in 2020

Curved fruit shape index in 2019

Curved fruit shape index in 2020

Fruit shape index internal in 2019

Fruit shape index internal in 2020

Proximal fruit blockiness in 2019

Proximal fruit blockiness in 2020

Distal fruit blockiness in 2019

Distal fruit blockiness in 2020

Fruit shape triangle in 2019

Fruit shape triangle in 2020

Eccentricity in 2019

Eccentricity in 2020

Proximal eccentricity in 2019

Proximal eccentricity in 2020


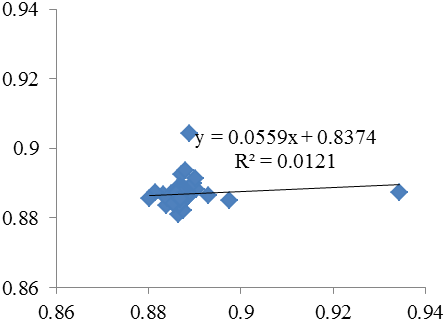

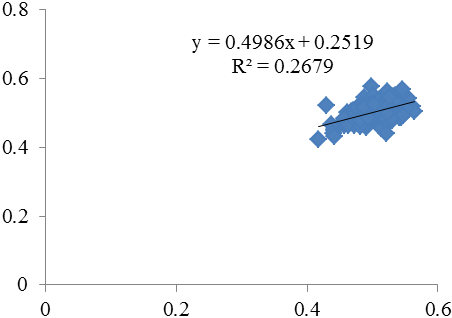

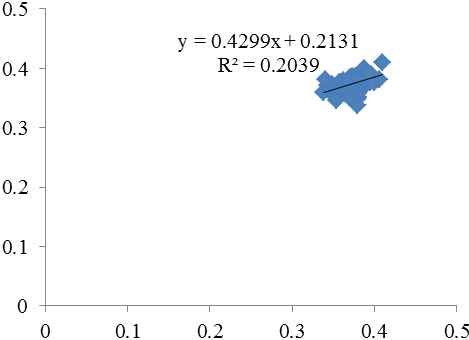

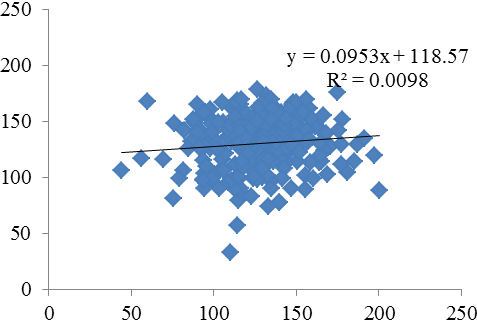

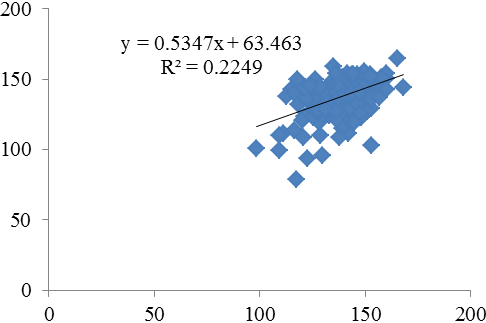

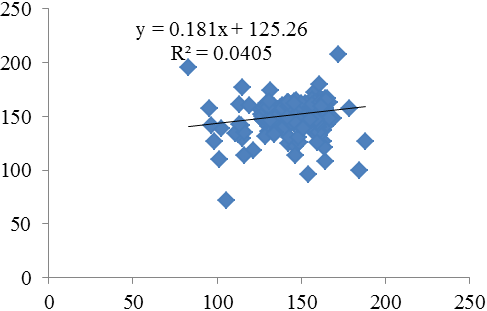

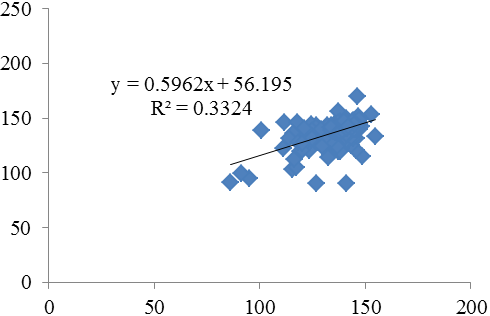

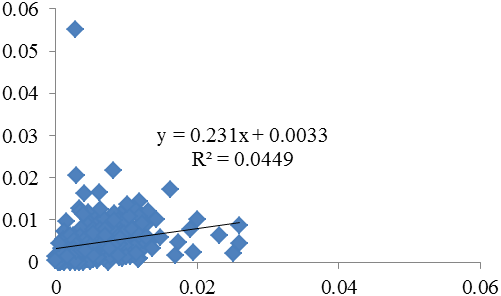


Distal eccentricity in 2019

Distal eccentricity in 2020

Width widest pos in 2019

Width widest pos in 2020

Eccentricity area index in 2019

Eccentricity area index in 2020

Proximal angle micro in 2019

Proximal angle micro in 2020

Proximal angle macro in 2019

Proximal angle macro in 2020

Distal angle micro in 2019

Distal angle micro in 2019

Distal angle macro in 2019

Distal angle macro in 2020

Proximal indentation area in 2019

Proximal indentation area in 2020


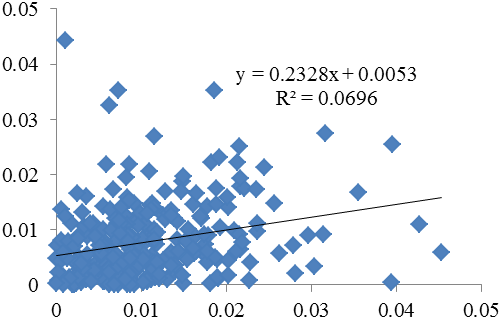


Shoulder height in 2019

Shoulder height in 2020

Fig. S4. Correlation analysis of the same berry shape-related traits across two years
