# Supplementary material for: Genome-wide association study of the candidate genes for grape berry shape-related traits
Source: BMC Plant Biol. 2022 Jan 20;22:42. doi: 10.1186/s12870-022-03434-x (PMC8772106; doi:10.1186/s12870-022-03434-x)
Supplement: Supplementary file 6 — Additional file 6: Table S2. SNP number distribution on each chromosome. [file 12870_2022_3434_MOESM6_ESM.docx]

Table S2. SNP number distribution on each chromosome

| Chromosome ID | Chromosome length / bp | SNP number | SNP density / bp |
| --- | --- | --- | --- |
| 1 | 23,037,639 | 30,060 | 765 |
| 2 | 18,779,844 | 21,213 | 884 |
| 3 | 19,341,862 | 26,393 | 730 |
| 4 | 23,867,706 | 29,151 | 817 |
| 5 | 25,021,643 | 36,145 | 690 |
| 6 | 21,508,407 | 26,035 | 824 |
| 7 | 21,026,613 | 26,156 | 802 |
| 8 | 22,385,789 | 29,510 | 757 |
| 9 | 23,006,712 | 31,390 | 731 |
| 10 | 18,140,952 | 25,639 | 705 |
| 11 | 19,818,926 | 26,210 | 754 |
| 12 | 22,702,307 | 33,237 | 681 |
| 13 | 24,396,255 | 33,299 | 731 |
| 14 | 30,274,277 | 41,811 | 722 |
| 15 | 20,304,914 | 28,212 | 717 |
| 16 | 22,053,297 | 29,303 | 751 |
| 17 | 17,126,926 | 20,367 | 839 |
| 18 | 29,360,087 | 38,271 | 766 |
| 19 | 24,021,853 | 33,727 | 711 |
| Total | 426,176,009 | 566,129 | 753 |
