# Supplementary material for: Genome-wide association study of the candidate genes for grape berry shape-related traits
Source: BMC Plant Biol. 2022 Jan 20;22:42. doi: 10.1186/s12870-022-03434-x (PMC8772106; doi:10.1186/s12870-022-03434-x)
Supplement: Supplementary file 7 — Additional file 7: Table S3. The variation in different berry shape parameters. [file 12870_2022_3434_MOESM7_ESM.docx]

Table S3. The variation in different berry shape parameters

| Characteristic | Maximum | Minimum | Mean | Standard deviation | Coefficient of variation |
| --- | --- | --- | --- | --- | --- |
| Perimeter | 10.03 | 2.91 | 6.65 | 1.43 | 21.52% |
| Area | 6.91 | 0.60 | 3.05 | 1.23 | 40.24% |
| Width mid-height | 2.65 | 0.82 | 1.77 | 0.36 | 20.19% |
| Maximum width | 2.66 | 0.82 | 1.78 | 0.36 | 20.08% |
| Height mid-width | 3.66 | 0.91 | 2.06 | 0.49 | 23.87% |
| Maximum height | 3.71 | 0.93 | 2.08 | 0.50 | 23.86% |
| Curved height | 3.77 | 1.03 | 2.21 | 0.48 | 21.80% |
| Fruit shape index external I | 2.12 | 0.96 | 1.19 | 0.16 | 13.14% |
| Fruit shape index external II | 2.20 | 0.95 | 1.19 | 0.16 | 13.43% |
| Curved fruit shape index | 2.27 | 1.03 | 1.28 | 0.16 | 12.39% |
| Fruit shape index internal | 2.21 | 0.95 | 1.19 | 0.16 | 13.48% |
| Proximal fruit Blockiness | 1.45 | 0.77 | 0.89 | 0.09 | 10.18% |
| Distal fruit blockiness | 0.70 | 0.56 | 0.62 | 0.02 | 3.03% |
| Fruit shape triangle | 0.92 | 0.73 | 0.81 | 0.03 | 4.00% |
| Eccentricity | 1.06 | 0.77 | 0.90 | 0.04 | 4.04% |
| Proximal eccentricity | 0.84 | 0.81 | 0.84 | 0.00 | 0.36% |
| Distal eccentricity | 0.91 | 0.89 | 0.89 | 0.00 | 0.18% |
| Width eidest pos | 0.56 | 0.42 | 0.51 | 0.02 | 3.93% |
| Eccentricity area index | 0.41 | 0.35 | 0.37 | 0.01 | 2.00% |
| Proximal angle micro | 175.75 | 71.97 | 129.81 | 17.56 | 13.53% |
| Proximal angle macro | 165.01 | 98.06 | 139.76 | 9.09 | 6.51% |
| Distal angle micro | 189.69 | 88.89 | 151.36 | 11.46 | 7.57% |
| Distal angle macro | 158.27 | 88.49 | 137.44 | 8.48 | 6.17% |
| Proximal indentation area | 0.03 | 0.00 | 0.01 | 0.00 | 63.64% |
| Shoulder height | 0.03 | 0.00 | 0.01 | 0.01 | 63.58% |
