# Supplementary material for: Genome-wide association study of the candidate genes for grape berry shape-related traits
Source: BMC Plant Biol. 2022 Jan 20;22:42. doi: 10.1186/s12870-022-03434-x (PMC8772106; doi:10.1186/s12870-022-03434-x)
Supplement: Supplementary file 8 — Additional file 8: Table S4. Total variance explained. [file 12870_2022_3434_MOESM8_ESM.docx]

| **Table S4 Total variance explained** | | | | | | |
| --- | --- | --- | --- | --- | --- | --- |
| Component | Initial Eigenvalues | | | Extraction Sums of Squared Loadings | | |
|  | Total | % of Variance | Cumulative % | Total | % of Variance | Cumulative % |
| 1 | 8.557 | 34.228 | 34.228 | 8.557 | 34.228 | 34.228 |
| 2 | 5.131 | 20.525 | 54.752 | 5.131 | 20.525 | 54.752 |
| 3 | 2.472 | 9.887 | 64.640 | 2.472 | 9.887 | 64.640 |
| 4 | 2.032 | 8.129 | 72.769 | 2.032 | 8.129 | 72.769 |
| 5 | 1.414 | 5.657 | 78.426 | 1.414 | 5.657 | 78.426 |
| 6 | .964 | 3.857 | 82.283 |  |  |  |
| 7 | .937 | 3.749 | 86.032 |  |  |  |
| 8 | .869 | 3.475 | 89.507 |  |  |  |
| 9 | .724 | 2.898 | 92.404 |  |  |  |
| 10 | .633 | 2.532 | 94.937 |  |  |  |
| 11 | .340 | 1.360 | 96.296 |  |  |  |
| 12 | .300 | 1.198 | 97.494 |  |  |  |
| 13 | .268 | 1.071 | 98.566 |  |  |  |
| 14 | .131 | .523 | 99.089 |  |  |  |
| 15 | .110 | .441 | 99.530 |  |  |  |
| 16 | .056 | .224 | 99.754 |  |  |  |
| 17 | .033 | .131 | 99.885 |  |  |  |
| 18 | .017 | .068 | 99.953 |  |  |  |
| 19 | .005 | .022 | 99.974 |  |  |  |
| 20 | .004 | .016 | 99.991 |  |  |  |
| 21 | .002 | .007 | 99.997 |  |  |  |
| 22 | .001 | .002 | 99.999 |  |  |  |
| 23 | .000 | .000 | 100.000 |  |  |  |
| 24 | 1.917E-005 | 7.668E-005 | 100.000 |  |  |  |
| 25 | 1.205E-005 | 4.819E-005 | 100.000 |  |  |  |
| Extraction Method: Principal Component Analysis. | | | | | | |
