# Supplementary material for: Genome-wide association study of the candidate genes for grape berry shape-related traits
Source: BMC Plant Biol. 2022 Jan 20;22:42. doi: 10.1186/s12870-022-03434-x (PMC8772106; doi:10.1186/s12870-022-03434-x)
Supplement: Supplementary file 9 — Additional file 9: Table S5. Component matrix of grape berry shape-related parameters. [file 12870_2022_3434_MOESM9_ESM.docx]

| **Table S5 Component matrix^a^ of grape berry shape-related parameters** | | | | | |
| --- | --- | --- | --- | --- | --- |
| Traits | Component | | | | |
|  | 1 | 2 | 3 | 4 | 5 |
| Perimeter | .804 | .519 | -.080 | .232 | -.076 |
| Area | .790 | .583 | .090 | .103 | -.030 |
| Width mid-height | .583 | .788 | .065 | .139 | -.085 |
| Maximum width | .594 | .777 | .051 | .154 | -.091 |
| Height mid-width | .924 | .361 | .084 | .062 | -.018 |
| Maximum height | .925 | .356 | .074 | .074 | -.012 |
| Curved height | .905 | .369 | -.048 | .178 | -.045 |
| Fruit shape index external I | .814 | -.538 | .056 | -.093 | .107 |
| Fruit shape index external II | .814 | -.551 | .052 | -.080 | .074 |
| Curved fruit shape index | .675 | -.647 | -.236 | .150 | .042 |
| Fruit shape index internal | .813 | -.551 | .052 | -.080 | .075 |
| Proximal fruit blockiness | .755 | -.599 | .126 | -.034 | .043 |
| Distal fruit Blockiness | .107 | -.055 | .732 | .172 | .399 |
| Fruit Shape Triangle | -.062 | -.412 | .308 | .362 | -.456 |
| Eccentricity | -.381 | -.151 | .491 | .440 | -.343 |
| Proximal Eccentricity | .003 | .241 | .421 | -.439 | -.325 |
| Distal Eccentricity | .161 | -.093 | -.130 | .282 | -.165 |
| Width Widest Pos | .140 | .413 | -.477 | -.494 | .328 |
| Eccentricity Area Index | .155 | -.282 | .673 | .043 | .478 |
| Proximal Angle Micro | .057 | -.079 | -.260 | -.212 | -.296 |
| Proximal Angle Macro | -.593 | .445 | .360 | .148 | -.070 |
| Distal Angle Micro | .018 | .275 | .372 | -.286 | .197 |
| Distal Angle Macro | -.505 | .651 | .244 | -.197 | .234 |
| Proximal Indentation Area | -.295 | .097 | -.337 | .663 | .356 |
| Shoulder Height | -.357 | .081 | -.314 | .582 | .311 |
| a. 5 components extracted. | | | | | |
|  | | | | | |
