# Supplementary material for: Genome-wide association study of the candidate genes for grape berry shape-related traits
Source: BMC Plant Biol. 2022 Jan 20;22:42. doi: 10.1186/s12870-022-03434-x (PMC8772106; doi:10.1186/s12870-022-03434-x)
Supplement: Supplementary file 15 — Additional file 15: Table S11. Details of SNP loci associated with multiple berry-shape traits identified via GWAS from both years. [file 12870_2022_3434_MOESM15_ESM.docx]

Table S11. Details of SNP loci associated with multiple berry-shape traits identified via GWAS from both years

| Trait | Marker Name | Chr | Pos | Allele (MAF) | Func. Ref Gene | Gene. Ref. Gene | Gene Detail. Ref. Gene |
| --- | --- | --- | --- | --- | --- | --- | --- |
| Fruit shape index external I,  Fruit shape index external II | 17_604843 | 14 | 18869900 | 0.080 | intronic | *VIT_14s0006g01980* | . |
|  | 17_173984 | 7 | 7722025 | 0.060 | intergenic | *VIT_07s0005g04520*; *VIT_07s0005g04530* | dist=2184;dist=9350 |
| Fruit shape index external II,  Fruit shape index internal | 17_173101 | 7 | 7178080 | 0.051 | intergenic | *VIT_07s0005g04080*; *VIT_07s0005g04100* | dist=2043;dist=21176 |
|  | 17_174870 | 7 | 8218447 | 0.150 | intergenic | *VIT_07s0005g04950*; *VIT_07s0005g04970* | dist=5996;dist=24703 |
|  | 17_174873 | 7 | 8218474 | 0.152 | intergenic | *VIT_07s0005g04950*; *VIT_07s0005g04970* | dist=6023;dist=24676 |
|  | 17_183754 | 7 | 1.6E+07 | 0.112 | intronic | *VIT_07s0129g00250* | . |
|  | 17_192552 | 6 | 32966 | 0.135 | intergenic | *VIT_06s0004g00010*; *VIT_06s0004g00020* | dist=24850;dist=63028 |
|  | 17_194432 | 6 | 1511157 | 0.106 | intergenic | *VIT_06s0004g01270*; *VIT_06s0004g01280* | dist=1972;dist=12995 |
|  | 17_221952 | 16 | 181634 | 0.064 | intronic | *VIT_16s0039g00370* | . |
|  | 17_241778 | 16 | 1.3E+07 | 0.056 | intergenic | *VIT_16s0022g01190*; *VIT_16s0022g01210* | dist=9905;dist=50098 |
|  | 17_244898 | 16 | 1.5E+07 | 0.053 | intergenic | *VIT_16s0022g02320*; *VIT_16s0022g02330* | dist=9625;dist=34074 |
|  | 17_274811 | 8 | 1.5E+07 | 0.077 | exonic | *VIT_08s0007g00210* | . |
|  | 17_274853 | 8 | 1.5E+07 | 0.084 | intronic | *VIT_08s0007g00290* | . |
|  | 17_275023 | 8 | 1.5E+07 | 0.131 | intergenic | *VIT_08s0007g00440*; *VIT_08s0007g00460* | dist=1417;dist=8857 |
|  | 17_298329 | 12 | 7763169 | 0.054 | intergenic | *VIT_12s0134g00230*; *VIT_12s0134g00240* | dist=7805;dist=5634 |
|  | 17_298333 | 12 | 7763248 | 0.051 | intergenic | *VIT_12s0134g00230*; *VIT_12s0134g00240* | dist=7884;dist=5555 |
|  | 17_300875 | 12 | 9590062 | 0.068 | intronic | *VIT_12s0057g00880* | . |
|  | 17_301963 | 12 | 1E+07 | 0.059 | intronic | *VIT_12s0057g01450* | . |
|  | 17_326541 | 9 | 2741353 | 0.066 | intergenic | *VIT_09s0002g03020*; *VIT_09s0002g03030* | dist=1155;dist=36859 |
|  | 17_335227 | 9 | 9032382 | 0.083 | intergenic | *VIT_09s0002g08290*; *VIT_09s0002g08300* | dist=56872;dist=67363 |
|  | 17_339011 | 9 | 1.1E+07 | 0.114 | intergenic | *VIT_09s0096g00040*; *VIT_09s0096g00060* | dist=35522;dist=113545 |
|  | 17_344244 | 9 | 14639734 | 0.051 | intergenic | *VIT_09s0070g00840*; *VIT_09s0070g00850* | dist=10608;dist=17357 |
|  | 17_391916 | 4 | 1731482 | 0.059 | intergenic | *VIT_04s0008g02120*; *VIT_04s0008g02140* | dist=4916;dist=12926 |
|  | 17_391923 | 4 | 1731636 | 0.059 | intergenic | *VIT_04s0008g02120*; *VIT_04s0008g02140* | dist=5070;dist=12772 |
|  | 17_402449 | 4 | 1E+07 | 0.075 | intergenic | *VIT_04s0079g00140*; *VIT_04s0079g00160* | dist=6968;dist=64234 |
|  | 17_40909 | 10 | 1.1E+07 | 0.252 | intergenic | *VIT_10s0092g00180*; *VIT_10s0092g00200* | dist=21029;dist=57259 |
|  | 17_414876 | 4 | 1.9E+07 | 0.068 | intronic | *VIT_04s0023g01970* | . |
|  | 17_423120 | 19 | 568811 | 0.218 | intergenic | *VIT_19s0014g00550*; *VIT_19s0014g00560* | dist=4768;dist=3906 |
|  | 17_423121 | 19 | 568813 | 0.061 | intergenic | *VIT_19s0014g00550*; *VIT_19s0014g00560* | dist=4770;dist=3904 |
|  | 17_443865 | 19 | 1.4E+07 | 0.062 | intergenic | *VIT_19s0138g00140*; *VIT_19s0135g00010* | dist=68643;dist=336059 |
|  | 17_501430 | 5 | 4184456 | 0.080 | intronic | *VIT_05s0020g02510* | . |
|  | 17_502215 | 5 | 4663676 | 0.120 | intronic | *VIT_05s0020g02930* | . |
|  | 17_502217 | 5 | 4663684 | 0.080 | intronic | *VIT_05s0020g02930* | . |
|  | 17_514579 | 5 | 1.2E+07 | 0.057 | intergenic | *VIT_05s0051g00940*; *VIT_05s0051g00970* | dist=48020;dist=54582 |
|  | 17_514582 | 5 | 1.2E+07 | 0.059 | intergenic | *VIT_05s0051g00940*; *VIT_05s0051g00970* | dist=48053;dist=54549 |
|  | 17_524353 | 5 | 1.8E+07 | 0.141 | intergenic | *VIT_05s0029g01530*; *VIT_05s0029g01540* | dist=15313;dist=11816 |
|  | 17_556821 | 18 | 1.4E+07 | 0.115 | intergenic | *VIT_18s0166g00210*; *VIT_18s0166g00220* | dist=7566;dist=25298 |
|  | 17_574526 | 18 | 2.7E+07 | 0.060 | upstream | *VIT_18s0041g02350* | dist=433 |
|  | 17_574527 | 18 | 2.7E+07 | 0.064 | upstream | *VIT_18s0041g02350* | dist=452 |
|  | 17_581049 | 14 | 2182095 | 0.072 | intergenic | *VIT_14s0060g02480*; *VIT_14s0060g02520* | dist=33710;dist=79072 |
|  | 17_587402 | 14 | 6702694 | 0.075 | intronic | *VIT_14s0030g01820* | . |
|  | 17_589156 | 14 | 8007929 | 0.056 | intergenic | *VIT_14s0081g00280*; *VIT_14s0081g00310* | dist=37125;dist=19068 |
|  | 17_600790 | 14 | 1.6E+07 | 0.065 | upstream | *VIT_14s0006g01170* | dist=662 |
|  | 17_86342 | 3 | 7842466 | 0.064 | intronic | *VIT_03s0091g01190* | . |
|  | 17_89302 | 3 | 9892726 | 0.095 | upstream | *VIT_03s0097g00130*; *VIT_03s0097g00140* | dist=46 |
|  | 17_94504 | 3 | 12918968 | 0.105 | exonic | *VIT_03s0110g00170* | . |
| Curved fruit shape index,  Fruit shape index external II,  Fruit shape index internal | 17_173150 | 7 | 7207450 | 0.059 | intronic | *VIT_07s0005g04110* | . |
|  | 17_414350 | 4 | 1.8E+07 | 0.117 | intergenic | *VIT_04s0023g01620*; *VIT_04s0023g01630* | dist=8465;dist=9589 |
|  | 17_43912 | 10 | 1.3E+07 | 0.058 | upstream | *VIT_10s0042g00450* | dist=85 |
|  | 17_584380 | 14 | 4394619 | 0.074 | intergenic | *VIT_14s0030g00490*; *VIT_14s0030g00500* | dist=25236;dist=5675 |
| Fruit shape index external I,  Fruit shape index external II,  Fruit shape index internal | 17_10781 | 17 | 1E+07 | 0.051 | intronic | *VIT_17s0000g08830* | . |
|  | 17_13125 | 17 | 1.2E+07 | 0.052 | intergenic | *VIT_17s0000g09920*; *VIT_17s0000g09930* | dist=13342;dist=22751 |
|  | 17_169855 | 7 | 4739004 | 0.106 | intergenic | *VIT_07s0005g02370*; *VIT_07s0005g02380* | dist=8969;dist=4361 |
|  | 17_175993 | 7 | 9281757 | 0.063 | intergenic | *VIT_07s0005g05270*; *VIT_07s0005g05300* | dist=14398;dist=5427 |
|  | 17_23410 | 10 | 294861 | 0.051 | intergenic | *VIT_10s0116g00620*; *VIT_10s0116g00680* | dist=27611;dist=5756 |
|  | 17_27716 | 10 | 2996782 | 0.088 | intergenic | *VIT_10s0003g01580*; *VIT_10s0003g01590* | dist=21234;dist=3546 |
|  | 17_27724 | 10 | 2997079 | 0.091 | intergenic | *VIT_10s0003g01580*; *VIT_10s0003g01590* | dist=21531;dist=3249 |
|  | 17_288257 | 12 | 1460924 | 0.073 | intergenic | *VIT_12s0028g00940*; *VIT_12s0028g00960* | dist=10479;dist=6597 |
|  | 17_296302 | 12 | 6656303 | 0.09 | upstream | *VIT_12s0059g01800* | dist=171 |
|  | 17_300474 | 12 | 9258980 | 0.051 | intergenic | *VIT_12s0057g00610*; *VIT_12s0057g00620* | dist=53445;dist=7371 |
|  | 17_300861 | 12 | 9588653 | 0.059 | intronic | *VIT_12s0057g00880* | . |
|  | 17_300862 | 12 | 9588679 | 0.059 | intronic | *VIT_12s0057g00880* | . |
|  | 17_300864 | 12 | 9588935 | 0.06 | intronic | *VIT_12s0057g00880* | . |
|  | 17_308271 | 12 | 1.40E+07 | 0.08 | intergenic | *VIT_12s0055g00740*; *VIT_12s0055g00750* | dist=42642;dist=7287 |
|  | 17_308635 | 12 | 1.40E+07 | 0.06 | intergenic | *VIT_12s0055g00820*; *VIT_12s0055g00830* | dist=19155;dist=9976 |
|  | 17_3269 | 17 | 3234391 | 0.094 | intergenic | *VIT_17s0000g03350*; *VIT_17s0000g03360* | dist=1833;dist=1307 |
|  | 17_335474 | 9 | 9172296 | 0.063 | intergenic | *VIT_09s0002g08350*; *VIT_09s0002g08370* | dist=17731;dist=74144 |
|  | 17_335515 | 9 | 9199661 | 0.057 | intergenic | *VIT_09s0002g08350*; *VIT_09s0002g08370* | dist=45096;dist=46779 |
|  | 17_335953 | 9 | 9377425 | 0.061 | intergenic | *VIT_09s0002g08390*; *VIT_09s0002g08400* | dist=33064;dist=8348 |
|  | 17_336078 | 9 | 9440506 | 0.111 | intergenic | *VIT_09s0002g08430*; *VIT_09s0002g08440* | dist=5977;dist=6870 |
|  | 17_337117 | 9 | 9975680 | 0.079 | intergenic | *VIT_09s0002g08700*; *VIT_09s0002g08710* | dist=15750;dist=13841 |
|  | 17_34010 | 10 | 7376378 | 0.053 | intergenic | *VIT_10s0003g04290*; *VIT_10s0003g04300* | dist=7820;dist=17891 |
|  | 17_34014 | 10 | 7376556 | 0.051 | intergenic | *VIT_10s0003g04290*; *VIT_10s0003g04300* | dist=7998;dist=17713 |
|  | 17_34015 | 10 | 7376558 | 0.051 | intergenic | *VIT_10s0003g04290*; *VIT_10s0003g04300* | dist=8000;dist=17711 |
|  | 17_340457 | 9 | 1.30E+07 | 0.113 | intergenic | *VIT_09s0096g00840*; *VIT_09s0096g00850* | dist=16696;dist=8826 |
|  | 17_340496 | 9 | 1.30E+07 | 0.057 | intergenic | *VIT_09s0096g00840*; *VIT_09s0096g00850* | dist=23266;dist=2256 |
|  | 17_340787 | 9 | 1.30E+07 | 0.153 | intergenic | *VIT_09s0070g00010*; *VIT_09s0070g00020* | dist=36534;dist=15032 |
|  | 17_341428 | 9 | 1.30E+07 | 0.092 | intergenic | *VIT_09s0070g00140*; *VIT_09s0070g00150* | dist=32792;dist=28128 |
|  | 17_352058 | 9 | 2.00E+07 | 0.061 | intergenic | *VIT_09s0018g02060*; *VIT_09s0018g02070* | dist=45554;dist=38445 |
|  | 17_368027 | 1 | 7723053 | 0.091 | downstream | *VIT_01s0127g00380* | dist=411 |
|  | 17_443865 | 19 | 1.40E+07 | 0.062 | intergenic | *VIT_19s0138g00140*; *VIT_19s0135g00010* | dist=68643;dist=336059 |
|  | 17_447676 | 19 | 1.70E+07 | 0.052 | exonic | *VIT_19s0093g00140* | . |
|  | 17_44954 | 10 | 1.40E+07 | 0.11 | intergenic | *VIT_10s0042g00860*; *VIT_10s0042g00870* | dist=10552;dist=18684 |
|  | 17_44955 | 10 | 1.40E+07 | 0.11 | intergenic | *VIT_10s0042g00860*; *VIT_10s0042g00870* | dist=10727;dist=18509 |
|  | 17_44958 | 10 | 1.40E+07 | 0.11 | intergenic | *VIT_10s0042g00860*; *VIT_10s0042g00870* | dist=10752;dist=18484 |
|  | 17_469703 | 13 | 6980839 | 0.062 | intronic | *VIT_13s0019g05160* | . |
|  | 17_512984 | 5 | 1.10E+07 | 0.097 | intronic | *VIT_05s0051g00580* | . |
|  | 17_52489 | 2 | 1302967 | 0.055 | intergenic | *VIT_02s0025g01360*; *VIT_02s0025g01370* | dist=27758;dist=7015 |
|  | 17_552368 | 18 | 1.10E+07 | 0.064 | exonic | *VIT_18s0001g12960* | . |
|  | 17_559777 | 18 | 1.60E+07 | 0.108 | intronic | *VIT_18s0076g00330* | . |
|  | 17_580198 | 14 | 1670055 | 0.065 | intergenic | *VIT_14s0060g02080*; *VIT_14s0060g02090* | dist=3081;dist=3977 |
|  | 17_6292 | 17 | 6610856 | 0.075 | intergenic | *VIT_17s0000g06030*; *VIT_17s0000g06040* | dist=3477;dist=2464 |
|  | 17_86347 | 3 | 7842721 | 0.056 | intronic | *VIT_03s0091g01190* | . |
| Curved fruit shape index,  Fruit shape index external I,  Fruit shape index external II,  Fruit shape index internal, | 17_103635 | 11 | 56558 | 0.050 | intronic | *VIT_11s0016g00100* | . |
|  | 17_158117 | 15 | 16652316 | 0.064 | intergenic | *VIT_15s0048g02460*; *VIT_15s0048g02480* | dist=5875;dist=4378 |
|  | 17_165943 | 7 | 1909757 | 0.091 | intronic | *VIT_07s0104g00760* | . |
|  | 17_165944 | 7 | 1909812 | 0.092 | intronic | *VIT_07s0104g00760* | . |
|  | 17_165946 | 7 | 1909841 | 0.092 | intronic | *VIT_07s0104g00760* | . |
|  | 17_165948 | 7 | 1910109 | 0.092 | intronic | *VIT_07s0104g00760* | . |
|  | 17_165952 | 7 | 1911848 | 0.094 | intronic | *VIT_07s0104g00760* | . |
|  | 17_173305 | 7 | 7304129 | 0.174 | intronic | *VIT_07s0005g04190* | . |
|  | 17_176706 | 7 | 9741085 | 0.054 | intergenic | *VIT_07s0005g05600*; *VIT_07s0005g05610* | dist=9984;dist=26663 |
|  | 17_211324 | 6 | 14073766 | 0.054 | intronic | *VIT_06s0009g01890* | . |
|  | 17_230957 | 16 | 6407092 | 0.070 | intronic | *VIT_16s0013g00860* | . |
|  | 17_304846 | 12 | 11982054 | 0.063 | intergenic | *VIT_12s0121g00130*; *VIT_12s0121g00180* | dist=74962;dist=45305 |
|  | 17_305195 | 12 | 12213794 | 0.051 | intergenic | *VIT_12s0121g00250*; *VIT_12s0121g00270* | dist=26142;dist=25724 |
|  | 17_309493 | 12 | 14463408 | 0.073 | intergenic | *VIT_12s0055g00990*; *VIT_12s0055g01000* | dist=8442;dist=5180 |
|  | 17_312547 | 12 | 16881941 | 0.062 | upstream | *VIT_12s0034g01030* | dist=568 |
|  | 17_326935 | 9 | 3036270 | 0.101 | upstream | *VIT_09s0002g03350* | dist=799 |
|  | 17_335450 | 9 | 9158760 | 0.076 | intergenic | *VIT_09s0002g08350*; *VIT_09s0002g08370* | dist=4195;dist=87680 |
|  | 17_337013 | 9 | 9938553 | 0.074 | intergenic | *VIT_09s0002g08690*; *VIT_09s0002g08700* | dist=27602;dist=20424 |
|  | 17_337015 | 9 | 9938588 | 0.073 | intergenic | *VIT_09s0002g08690*; *VIT_09s0002g08700* | dist=27637;dist=20389 |
|  | 17_482077 | 13 | 15044600 | 0.129 | exonic | *VIT_13s0175g00110* | . |
|  | 17_491897 | 13 | 21880725 | 0.211 | intronic | *VIT_13s0064g00360* | . |
|  | 17_516416 | 5 | 13538149 | 0.068 | intergenic | *VIT_05s0136g00210*; *VIT_05s0136g00240* | dist=19698;dist=23208 |
|  | 17_528926 | 5 | 20785814 | 0.051 | intronic | *VIT_05s0124g00250* | . |
|  | 17_552659 | 18 | 11211922 | 0.053 | intronic | *VIT_18s0001g13160* | . |
|  | 17_572735 | 18 | 26096613 | 0.158 | intergenic | *VIT_18s0041g01360*; *VIT_18s0041g01370* | dist=19622;dist=16356 |
|  | 17_590009 | 14 | 8600158 | 0.059 | intergenic | *VIT_14s0081g00500*; *VIT_14s0081g00510* | dist=12473;dist=55968 |
|  | 17_88457 | 3 | 9329092 | 0.050 | intergenic | *VIT_03s0088g01080*; *VIT_03s0088g01090* | dist=14963;dist=11064 |
|  | 17_88457 | 3 | 9329092 | 0.050 | intergenic | *VIT_03s0088g01080*; *VIT_03s0088g01090* | dist=14963;dist=11064 |
|  | 17_88459 | 3 | 9329303 | 0.050 | intergenic | *VIT_03s0088g01080*; *VIT_03s0088g01090* | dist=15174;dist=10853 |

Chr: chromosome; Pos: position; MAF: minor allele frequency; Func. Ref. Gene: The functional region of the reference gene in which the mutation is located. Gene. Ref. Gene: the name of the reference gene of the mutation (if it is between genes, it is the genes on both sides); Gene Detail. Ref. Gene: the non-exon region is located in a specific position in a specific transcript (if it is between genes, it is the distance from the genes on both sides).
